# Long-term returns to local health-care spending

**DOI:** 10.1007/s10198-024-01695-x

**Published:** 2024-05-18

**Authors:** Jakub Červený, Jan C. van Ours

**Affiliations:** 1 Institute for Health Care Analyses, Ministry of Health of the Slovak Republic, Bratislava, Slovakia; 2 National Health Information Center, Bratislava, Slovakia; 3 Institute of Economic Research, Slovak Academy of Sciences, Bratislava, Slovakia; 4 Erasmus School of Economics, Erasmus University, Rotterdam, The Netherlands; 5 Tinbergen Institute, Amsterdam/Rotterdam, The Netherlands; 6 CEPR, London, UK

**Keywords:** Health-care spending, Heart attack, Mortality, Duration models, C41, H75, I11, I18

## Abstract

This paper investigates the effects of health-care spending on mortality rates of patients who experienced a heart attack. We relate in-hospital deaths to in-hospital spending and post-discharge deaths to post-discharge health-care spending. In our analysis, we use detailed administrative data on individual personal characteristics including comorbidities, information about the type of medical treatment and information about health-care expenses at the regional level. To account for potential selectivity in the region of health-care treatment we compare local patients with visitors and stayers with recent movers from a different region. We find that in regions with higher health-care spending mortality after heart attacks is substantially lower. From this we conclude that there are long-term returns to local health-care spending.

## Introduction

Regional variations in health-care spending are often associated with regional differences in health outcomes, negatively or positively. The negative association may reflect a causal relationship from health-care expenditures to outcomes, a positive correlation may be driven by the underlying health status of regional populations, i.e. unhealthier populations require more health-care spending. It is also possible that there is no observable association because both effects cancel each other out.

This paper aims to establish whether there is a causal relationship between in-hospital and post-discharge health-care spending and health outcomes. We measure health-care spending at the regional level and health outcomes at the level of individual patients. In order to establish causality we take into account the effects of various other potential determinants of health outcomes: individual characteristics like age, gender, health status and unobserved individual characteristics such as vulnerability to certain health shocks. Furthermore, we have to take into account that the choice of patients for certain hospitals may affect their health outcome. Finally, we have to account for possible reverse causality, i.e., regions with an unhealthy population spend more on health care. To rule out that the choice of patients for specific hospitals influences health outcomes we focus on heart attacks, i.e. acute myocardial infarctions (AMI). Individuals who suffer from a heart attack need urgent health care quickly and will therefore be taken to the nearest catheterization center.^1^

Our individual-level data provide information about in-hospital mortality and mortality after being discharged from the hospital, as well as information about in-hospital and post-discharge health-care costs.^2^ Some studies use as part of their identification strategy the distance to hospital [2–5], other studies compare local patients with visitors, exploit reforms affecting patients’ choice of hospitals or study recently migrated patients [6]. To account for potential reverse causality related to in-hospital spending we compare heart attacks of locals—patients who were hospitalized in their region of residence, and heart attacks of visitors, i.e. patients who were hospitalized in a different region because they visited that area while experiencing a heart attack [7]. Similarly, to account for potential reverse causality in post-discharge spending, we use information on patients migrating between regions before they experienced a heart attack. Using information on visitors or migrants as part of our identification strategy is appealing. Local populations may represent a selective sample due to the fact that local-area health-care spending reflects the underlying observed and unobserved health status of the inhabitants. Several studies document the relationship between higher treatment costs and mortality, suggesting that patients in worse health require more intense treatments. Even though the risk factors for heart attacks are relatively well-known, their occurrence is not foreseeable. Heart attacks represent unanticipated health shocks and therefore should not affect the travel decisions of visitors. Thus, being a visitor or a regional migrant should be plausibly exogenous to the local-area health-care spending and treatment intensity. In addition to using the difference between locals and visitors and stayers and movers we also investigated within-group differences by estimating transition rate models (of mortality, hospital discharge and in-hospital mortality) that allow for the inclusion of observed and unobserved heterogeneity affecting outcomes. We find that in each transition rate there is unobserved heterogeneity. So, conditional on the observed characteristics and the elapsed duration there are unobserved characteristics influencing each transition rate. However, when we take the correlation between these unobserved characteristics across the transition processes into account the main parameter estimates do not change. In other words, there are no unobserved differences between individuals that could cause a spurious relationship between health-care spending and mortality.

Our analysis contributes to the literature in several ways. First, whereas previous studies are limited to in-hospital mortality we also study post-hospital-discharge mortality. Mortality is still high following the discharge from hospital. Therefore, focusing only on in-hospital costs and mortality will not provide the full answer to the question whether more intense treatments provide better health outcomes. To our knowledge, our paper is the first to investigate the causal effects of post-discharge care on survival from a heart attack. Second, our analysis takes into account potential selectivity in both types of health-care expenditures. For in-hospital health care we compare locals with visitors. For post-discharge health care we compare stayers and movers. If the relationship between expenses and mortality is similar for both pairs of patients selectivity is not an issue. Third, compared to usual regression techniques modeling mortality after a specific time interval, we use more sophisticated models that take into account the variation of the mortality rates over the time elapsed since the heart attack. Furthermore, our econometric strategy allows for both observed and unobserved individual patient characteristics affecting in-hospital mortality, discharge and transfer from hospital, as well as post-discharge mortality. The traditional approach of modeling mortality rates assumes that post-discharge mortality is independent of in-hospital stay. In reality, however, hospital length of stay might be correlated with both in-hospital mortality, hospital discharge and transfer from hospital, while realization of each duration may have an effect on long-term mortality after discharge.

The remainder of the paper is organized as follows: the next section presents a brief overview of previous economic studies on the relationship between health-care expenditures and mortality following a heart attack. These studies are from different countries and time periods using a variety of identification strategies to take into account local supply factors, i.e., health-care expenditures being determined by demographic circumstances. Some studies use as part of their identification strategy the distance to hospital, other studies compare local patients with visitors, study recently migrated patients or exploit reforms affecting patients’ choice of hospitals. The studies differ in terms of conclusions with respect to the relationship between health-care expenditures and mortality. The third section presents our dataset providing descriptive statistics about our main variables in the analysis. We show that there is substantial cross-regional variation both in in-hospital and post-discharge health-care expenditures. Across regions both types of expenditures are positively correlated. We also provide information about the four types of patients we pairwise distinguish in our analysis, i.e. locals–visitors and stayers–movers. We show that the cross-regional differences in health-care spending are not correlated with cross-regional difference in age or comorbidities.

In the fourth section, we present our empirical analysis. We start with discussing the way we use duration information. We model the duration of in-hospital stay, distinguishing between three ways in which in-hospital stay can end: discharge, transfer and mortality. We also investigate the post-discharge duration until mortality. Using a multivariate mixed proportional hazard framework we allow residence characteristics as well as observed and unobserved personal characteristics to influence the four types of durations. By allowing for correlation between the unobserved characteristics, the models take into account potential unobserved selectivity in hospital dismissals. Our main finding is that higher health-care expenditures reduce mortality. The fifth section explores the mechanisms through which higher health-care expenditures reduce mortality. Finally, the sixth section summarizes our main findings. We conclude that mortality depends on age of the patient, the way the heart attack was treated in hospital, residence characteristics, comorbidities and the elapsed time period since the heart attack occurred. Our main conclusion is that the substantial regional variation in mortality is very much related to regional variation in health-care expenses.

Contrary to previous studies, which usually focus on mortality in the first days and weeks after the heart attack occurred we also study mortality after hospital discharge for over a year after the heart attack. Although from a lifetime perspective a year is short, compared to days and weeks it is a long-term perspective.

## Previous studies on the medical treatment heart attacks

When studying the effectiveness of medical treatment of a heart attack one has to consider possible endogeneity of the treatment, i.e., depending on the seriousness of the heart attack a particular treatment will be used. If some treatments are only used with a severe heart attack one might erroneously conclude that this treatment is not effective while conditional of the severity of the heart attack one might draw a different conclusion. Furthermore, one has to consider the possibility that there are unobserved characteristics of patients that affect both treatment and outcome. Patients with poor health may receive a different treatment than patient in good health. If the treatment is correlated with a higher mortality one might erroneously conclude that this is due to the treatment while in fact it may be related to the poor health status of the patient. To account for endogeneity and unobserved patient characteristics often an instrumental variable approach is used. A popular instrumental variable is the distance to a particular hospital because this distance is likely to determine the nature of the treatment while not directly influencing the health outcome of the treatment. Heart attacks are frequently studied because the severity of the negative health shock will force the use of the nearest hospital.^3^ This rules out the possibility that a particular hospital is chosen for example because of its reputation. To account for spurious cross-regional correlation between health-care spending and health outcomes information about visitors suffering from a heart attack may be used. Because of the urgency of the treatment visitors will not be transferred to their region of residence. Studying patients who got a heart attack when visiting a different region will therefore correct for the potential correlation between regional health status of the population and regional health-care expenditures.

There are quite a few studies that use an instrumental variable approach. McClellan et al. [2] for example analyze 4-year survival rates of U.S. patients after a heart attack using the distance of patients to particular hospitals as instrumental variables to account for unobserved characteristics and endogeneity of treatment types. They conclude that admissions of patients into hospitals treating more AMI patients translates into a decrease of mortality by less than one percent, after taking into account access to possible treatments. The authors also note that treatments administered within the first 24 h after admission provide the best long-term survival probability. Frances et al. [3] study whether mortality after a heart attack is influenced by whether or not a cardiologist is involved in the treatment. To account for selectivity in the assignment of a patient to a cardiologist an instrumental variable approach is used based on the difference in distance from a patient’s home to the nearest cardiologist hospital and the nearest non-cardiologist hospital. Their main conclusion is that treatment by a cardiologist does not have a significant effect on mortality of heart attack patients. [8] study the effect of government-induced initiated competition in the UK health-care market using death rates after treatment for heart attacks as a measure for health-care efficiency. They find that greater competition has had a small negative effect on the quality of health-care, i.e., death rates after heart attacks increased. Cutler [4] analyzes U.S. data to study the benefits of revascularization procedures up to 17 years after a heart attack. To account for selectivity the instrumental variable used is the differential distance to a hospital capable of providing revascularization. The main conclusion is that revascularization is highly effective in reducing mortality after a heart attack. Sanwald and Schober [5] analyze survival rates of heart attack patients in Austria. They focus on the effects of patients being initially admitted to hospitals providing percutaneous coronary interventions (PCI). To select for selectivity they use as instrumental variable the distance between the patient residence and the nearest hospital providing PCI concluding that the use of PCI substantially reduces mortality following a heart attack.

Doyle [7] analyzes the relationship between health-care spending and health outcomes focusing on heart-related emergencies using a different identification strategy. Some areas spend more on health care because they have greater levels of intensive care services and higher staff-to-patient ratios. Doyle studies hospital discharges in the state of Florida finding that areas with a higher treatment intensity of patients with heart-related emergencies achieve better results in terms of reduced in-hospital mortality. To account for possible endogeneity, the analysis exploits information about visitors, i.e. patients experiencing a health shock when visiting Florida.

Moscelli et al. [9] use a policy change for identification. They study the effects of a relaxation of constraints on the choice of hospital in the English National Health Service investigating whether this affected mortality for three high volume emergency conditions with high mortality risks: heart attack, hip fracture and stroke. The idea of the reform was that greater choice would increase competition between hospitals and thus improve the quality of care. The authors find reduced mortality related to hip fractures, but no effects for heart attacks and strokes.

Chandra and Staiger [10] analyze a US dataset on patient survival following heart attack focusing on potential allocative inefficiencies in treatment decisions across hospitals. They find that variation in the choice of reperfusion treatment is partly related to differences in comparative advantages of hospitals in terms of the effectiveness of the treatment. They find evidence of allocative inefficiencies but this seems to be related to hospital having imperfect information and a misperception of their comparative advantage rather than to medical malpractice.

Our paper follows [7] in the identification of the effects of health-care spending on in-hospital mortality by distinguishing locals and visitors. We have two additions to [7]. First, we also investigate the effects of post-hospital health-care spending on post-hospital mortality whereby the identification strategy is similar by comparing stayers and movers. Second, we estimate transition models that allow us to investigate the importance of selectivity within groups.

## Data and descriptives

### Characteristics of the dataset

**Fig. 1:**
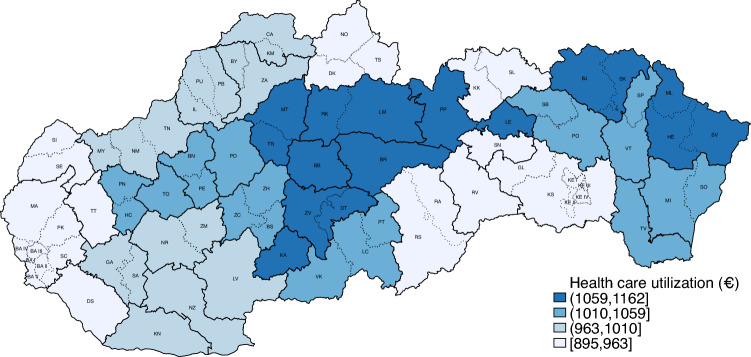
Health-Care utilization in Slovakia by Hospital Service Area; 2020 (Euro per patient). *Notes*: Solid border lines represent HSAs, dotted lines show district borders

Slovakia is a European country with about 5.5 million inhabitants of whom about half a million live in the capital Bratislava. Health-care expenditures in 2020 amounted to around 7.7% of GDP, which is slightly below the average OECD health-care spending of 9.9% of GDP. The 30-day mortality following admission to hospital with AMI is 13.5%, which is substantially higher than the OECD average of 8.8% (OECD [1]). Public health care in Slovakia is organized by hospital service area (HSA). HSAs in Slovakia are formally determined by government decree and include one or more neighboring districts, depending on the availability of acute care hospitals in the particular area. Figure 1 plots average health-care utilization by hospital service areas in 2020.^4^

An average HSA spends annually around 1017€ per patient on health care. There is a lot of regional variation. The majority of large cities, including Bratislava (BA) in the western part of the country and the second largest city of Košice (KE) in the eastern have an average utilization in the lowest quartile of the spending distribution. Both regions are the richest in terms of economic performance and have a dense network of specialist outpatient care and large university hospitals. The highest health-care utilization is present mostly in less populated rural areas across the country. This suggests that regional differences are likely related to the underlying health status of the local population.

Health care in Slovakia is based on universal coverage, with the national health insurance plan covering nearly all treatments, both inpatient, primary and secondary care as well as prescription medications. There are no co-payments for use of inpatient, primary or secondary care. Out-of-pocket payments mostly include procedures such as IVF, induced abortion, plastic surgery or above-standard accommodation.^5^

In our analysis, we use administrative data from the National Health Information Center of Slovakia (NHIC). NHIC administers several national health registries, including a claim database on all health expenditure reimbursements. The dataset combines patient-level data on all procedures provided by the national health insurance plan. The databases are linkable through a unique patient identifier obtained at birth. Health-care insurance is mandatory for every individual who has permanent residency in the Slovak Republic. Therefore the registries effectively cover the whole population.

Our sample includes all patients admitted to hospitals in the calendar years 2014–2019 with primary diagnosis code I21 and subcodes corresponding to AMI in the International Statistical Classification of Diseases (ICD) version 10. For each patient, the date of admission, discharge, length of stay, procedures, hospital charges,^6^ hospital performing the procedure and individual characteristics such as age, gender and residence are included. Residence characteristics including average educational attainment are based on data from the latest population census, while information about median income is based on 2020 data from the Social Security register. Patients in the dataset are observed until December 31, 2019. All observations beyond this date are considered as right-censored.^7^ Information on comorbidities is extracted from other registries including primary care procedures and pharmaceutical prescriptions. Following Bannay et al. [12] this information is used to construct the Charlson comorbidity index, according to an algorithm for administrative data developed by Quan et al. [13]. Because of the low occurrence of certain comorbidities such as AIDS or severe liver disease the original 17 categories of the index are collapsed into eight smaller categories. Our dataset is also informative about a variety of post-discharge treatments and procedures, such as the use of cardiac rehabilitation. This allows us to investigate possible mechanisms through which increased spending affects mortality.

In the analysis, we use two types of health-care spending as explanatory variables:

1. In-hospital spending $T_g$ calculated as follows: First, an average of total charges for each patient dying in a particular hospital is calculated (similar to Doyle [7]). Hospital averages are then aggregrated to the level of the HSA. More formally, the in-hospital spending or as it is sometimes referred to, treatment intensity *T* in HSA *g* is equal to:
  1 $$\begin{aligned} T_g=\frac{1}{H}\sum ^H_{h=1}\frac{1}{N}\sum ^N_{i=1} c_{ihg} \end{aligned}$$
  where *c* denotes total hospital charges for patient $i (1,\ldots ,N)$ dying at the hospital $h (1,\ldots ,H)$ in HSA *g*.
2. Post-discharge spending $T^P_g$ on patients surviving a heart attack is defined as the average of total costs billed in primary and secondary (specialized) care and for pharmaceutical treatments related to the ICD-10 diagnoses corresponding to AMI, for patients residing in a region *g* within the first 6 months after discharge from the hospital:
  2 $$\begin{aligned} T^P_g=\frac{1}{N}\sum _{i=1}^N c^P_{ig} \end{aligned}$$
  where $c^P_i$ denotes the costs for treatments within the first 6 months following discharge in HSA *g*.

**Fig. 2:**
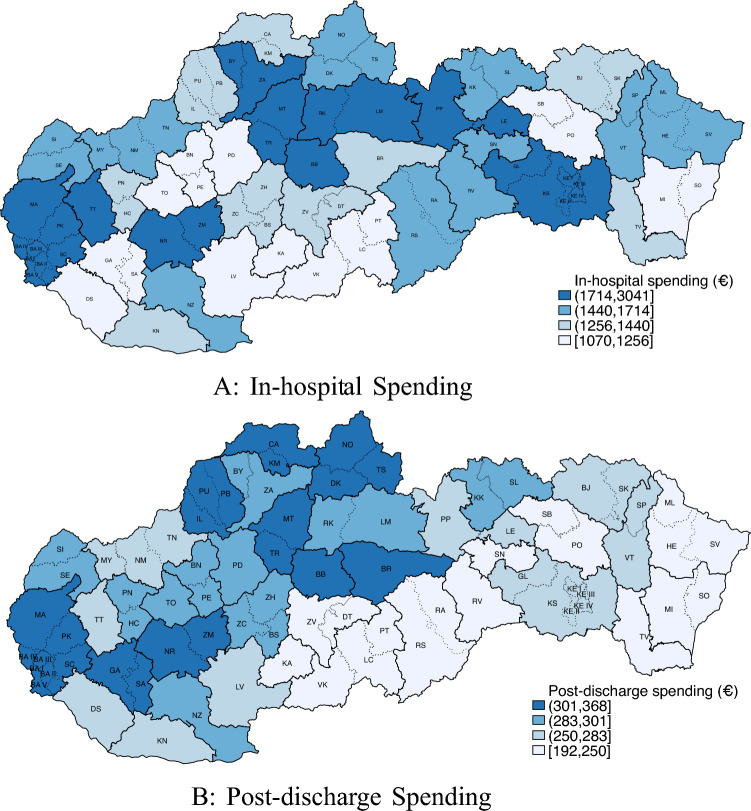
In-hospital and post-discharge spending in Slovakia by Hospital Service Area; 2020 (Euro per patient). *Notes*: Solid border lines represent HSA, dotted lines show district borders

The in-hospital spending reflects the treatment intensity of the hospital, as well as quality of the equipment, staff etc. The post-discharge spending reflects the quality and treatment intensity of outpatient services. Figure 2a, b provide an overview of HSAs and districts in Slovakia with their average treatment intensity. Not surprising, the highest spending areas are metropolitan areas, while lower spending areas are mostly rural.

### Visitors and movers

Information about the relationship between health-care spending and mortality of visitors and movers can be helpful in identifying the effects of health-care spending on mortality. Causal conclusions from the analysis presented rely on the assumption of random regional mobility of visitors and movers. This section provides evidence on the plausibility of this assumption.

Regional health-care expenditures may be high because of poor health of its inhabitants. If so, this will bias the estimated effect of expenditures on health outcomes. To investigate the relevance of this, we also analyze mortality of visitors with a heart attack. Patients with a confirmed AMI diagnosis are not always transferred to the nearest available hospital, but rather to a specialized PCI center—if such center is within 90 min of travel time. Therefore, a visitor is defined as a patient hospitalized with an AMI outside of their HSA, provided there is a cardiac center capable of performing PCI in patient’s home HSA.^8^ For patients with ST-elevated AMI and with no PCI center in their home HSA, we expand the catchment area to 90 min of travel time from their home municipality. In other words, we do not consider patients as visitors if they were hospitalised in a PCI center within 90 min of travel time from home, provided that there is no PCI center in their resident HSA. While visitors are helpful in the analysis of in-hospital spending on mortality, they are not informative about long-term mortality related to their home HSA once recovering from AMI. To establish a causal relationship between post-discharge spending and long-term mortality, we use information about patients who lived in a different region before experiencing a heart attack. To avoid potential selectivity we use the characteristics of the region of origin instead of their current region.

The use of visitors and movers as an identification strategy is particularly appealing in the analysis of regional variations in spending and mortality, since whether a patient is a visitor or a mover is unrelated to local spending at the HSA level, provided there is no systematic sorting to certain areas. A conceivable scenario which would invalidate causal inference of health-care spending levels on mortality is that areas spending more on health care attract wealthier visitors, who may be in better health overall. Similarly, certain areas with lower spending, concentrated mostly in rural areas may attract certain age cohorts.

**Fig. 3:**
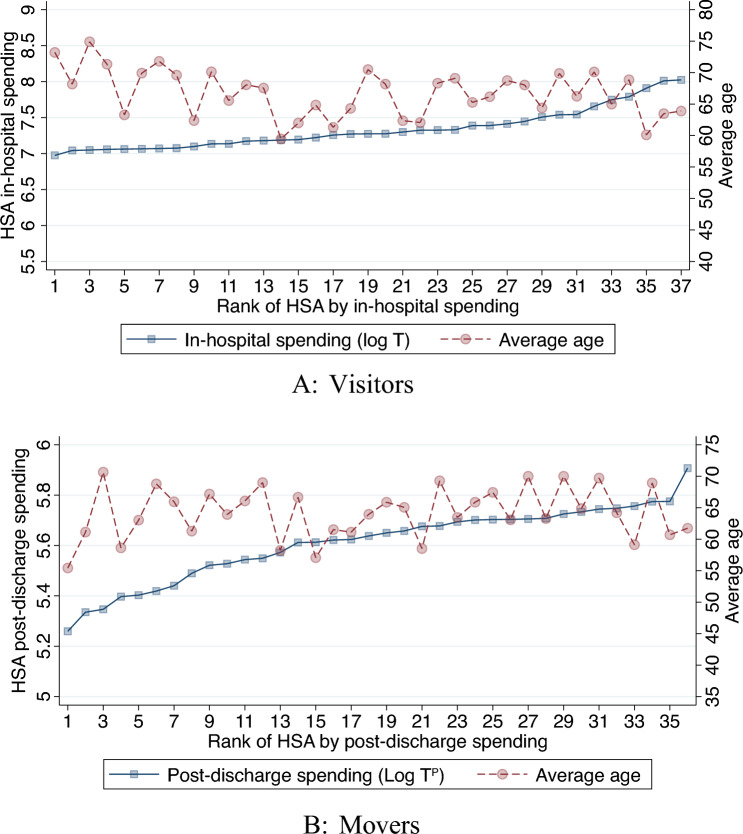
Age versus HSA spending rank

Figure 3 plots the average age of visitors and movers across the HSA spending distribution. For visitors, there is a noticeable decrease in age within the top 3 spending HSAs. For movers, there is no visible relationship. We also formally test the difference between the bottom quartile and the remaining quartiles. For visitors, the age decline is statistically significant when comparing the bottom and the top quartiles, while for movers there are no differences. To address these differences, all empirical analyses control for age of the patient, while in a sensitivity analysis we show the effects of health-care spending on mortality in separate age samples.

**Fig. 4:**
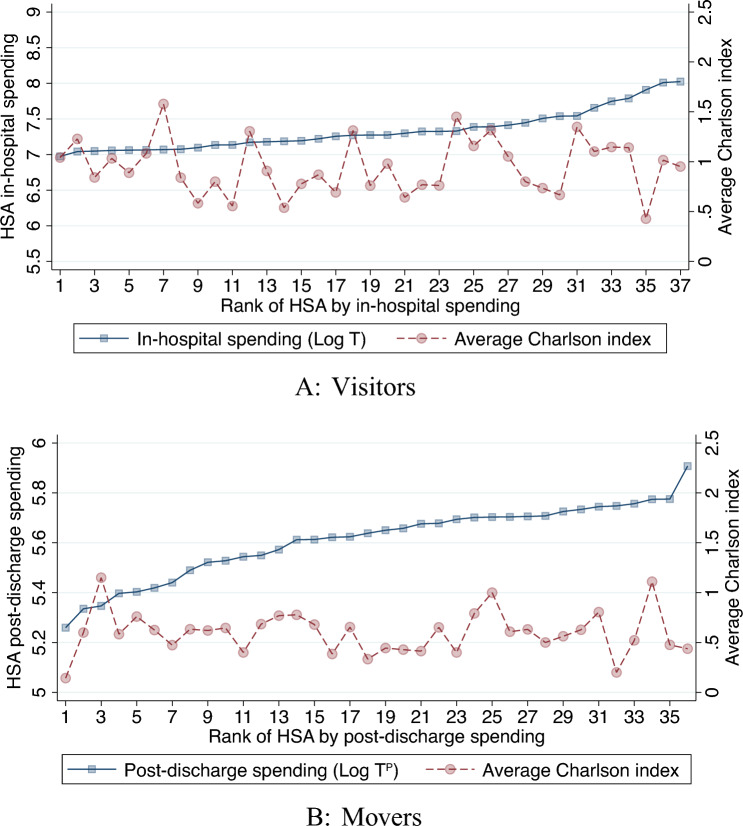
Charlson index versus HSA spending rank

To test whether certain HSAs attract patients with a different health status, a similar check is provided for Charlson comorbidity index in the respective HSA. As displayed in Fig. 4, for both groups there are no visible trends across the spending distribution. We also test for the differences in the share of patients with specific comorbidities across quartiles, again finding no significant differences except for a slightly higher incidence of cardiovascular disease for visitors in the third quartile and a slightly lower incidence of respiratory disease for movers when compared to the lowest quartile. Hospitals in the highest quartile of spending also have a slightly lower share of visitors admitted during weekends. This can be explained by the fact that these hospitals are located in the largest cities and centers of economic activity. During weekdays, there is a significant inflow of visitors—mostly commuters to work.^9^ The results are summarized in “Additional results and tables” of appendix in Tables 12 and 13.

**Fig. 5:**
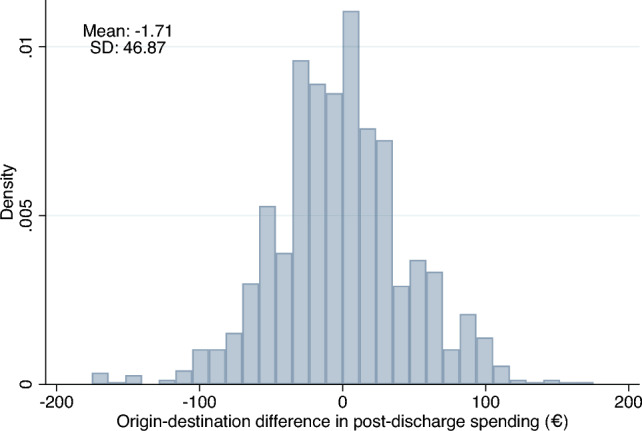
Origin–destination difference in HSA post-discharge spending

**Fig. 6:**
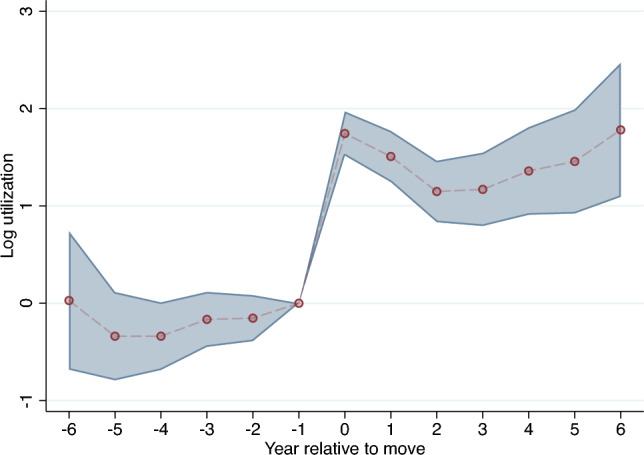
Estimated event study coefficients of health care utilization prior to move. *Notes*: Red dots represent estimated coefficients, shaded area displays 95% confidence intervals

Patients migrating between regions are only useful for identification of causal effects on post-discharge spending if the moves occur in both directions, i.e. patients move both from low-spending regions to high-spending regions and vice versa. To investigate whether this is the case, we follow Finkelstein et al. [15] and Godøy and Huitfeldt [6] and plot the distribution of differences in post-discharge spending between the origin and destination HSAs. As shown in Fig. 5 this distribution is fairly symmetrical. Another threat to this strategy is a possibility of movers choosing a high-spending region due to gradual worsening of their health status prior to heart attack. In principle, any systematic moving under this scenario should manifest as an increase in health care utilization in years prior to relocation. Figure 6 plots estimated event study coefficients of health care utilization in years prior to move, showing that there is no systematic trend.^10^

### Descriptive statistics

Table 1 provides summary statistics of the dataset, distinguished by quartiles of the distribution of health-care expenditures.^11^ Panel A shows the average (log) in-hospital costs over the four quartiles and selected sample characteristics for locals and visitors, while panel B provides similar information about post-hospital discharge health-care spending for the samples of stayers and movers.

Table 1 shows the distribution of two outcome variables, i.e. in-hospital mortality and post-discharge mortality. As shown in panel A, for both locals and visitors, in-hospital mortality is highest for the quartile with the lowest in-hospital expenditures. With respect to overall mortality also here for locals the lowest quartile of the in-hospital expenditures has the highest mortality. A similar relationship is present for visitors, where the lowest-spending quartile of HSAs has the highest in-hospital mortality. The same relationship holds for post-discharge mortality. Part of the inverse relationship between in-hospital spending and mortality may be due to differences in patient characteristics. For example, panel A shows that in the quartile with the highest in-hospital expenditures average age is lowest, both for locals and visitors. Furthermore, in this quartile among locals the value of the Charlson index is lowest. This also holds for visitors but here the differences are not statistically significant different from each other.

**Table 1:** Selected descriptive statistics by quartiles of health-care spending

	Q1	Q2	Q3	Q4	Q1	Q2	Q3	Q4
*Panel A: In-hospital mortality*								
	Locals				Visitors			
Health care spending								
In-hospital ($\log T_g$)	7.144	7.467*	7.809*	8.017*	7.176	7.444*	7.744*	8.019*
Sample characteristics								
In-hospital mortality	0.072	0.042	0.025*	0.043*	0.091	0.046*	0.036*	0.042*
Post-discharge mortality	0.308	0.247	0.233*	0.228*	0.300	0.269	0.267	0.237*
Age	69.0	67.5	66.5*	66.2*	67.6	66.2	65.8	63.7*
Charlson index	0.998	0.879	0.959	0.811*	0.980	0.873	1.091	0.978
Observations	10,840	10,227	9879	10,181	919	655	861	1086
*Panel B: Post-discharge mortality*								
	Stayers				Movers			
Health care spending								
Post-discharge ($\log T^P_g$)	5.434	5.624*	5.716*	5.790*	5.446	5.623*	5.719*	5.789*
Sample characteristics								
In-hospital mortality	0.043	0.054	0.054	0.034	0.023	0.022	0.017	0.027
Post-discharge mortality	0.254	0.274	0.258	0.236	0.203	0.196	0.182	0.203
Age	66.8	67.6	67.8*	66.8	64.1	62.9	65.6	64.0
Charlson index	0.936	0.960	0.888	0.894	0.816	0.692	0.799	0.872
Observations	12,390	11,819	12,014	7287	256	321	413	148

*Significantly different from bottom quartile at the 5-percent level. Standard errors clustered at the HSA level

Panel B of Table 1 provides similar descriptive statistics when the samples of stayers and movers are split-up by quartile of post-discharge health-care expenditures. Now, there is no clear relationship between health-care expenditures and mortality. There are also hardly any differences by quartile of post-discharge health-care expenditures and average age or Charlson index.

## Empirical analysis

### Modeling mortality rates

**Fig. 7:**
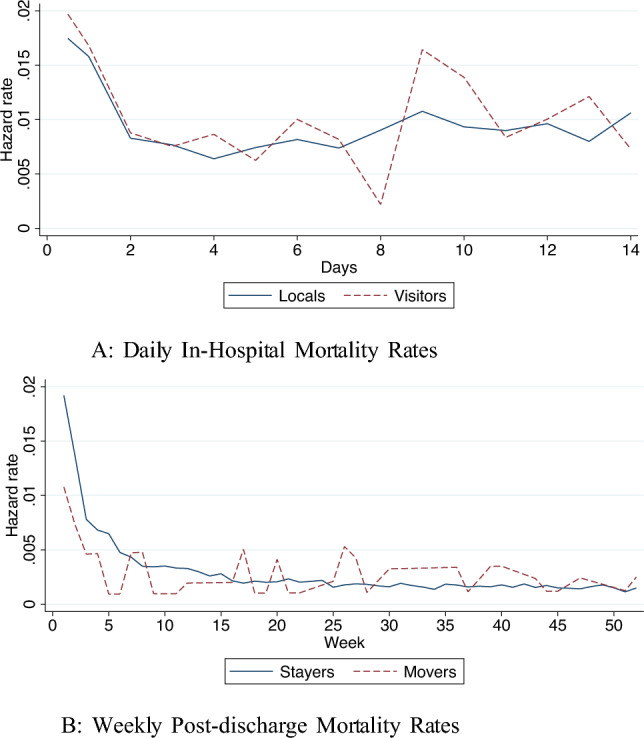
Empirical mortality rates after heart attack

Figure 7 presents empirical hazard rates of in-hospital mortality and mortality after discharge for both visitors and locals. Figure 7a plots daily in-hospital mortality rates for the first 14 days after being admitted to a hospital with a heart attack. The hazard rate peaks the first few days after admission, and then rises again over the course of the observation window, suggesting that more severe cases are kept longer in hospital. Figure 7b displays mortality rates after discharge from hospital. The mortality rate peaks shortly after discharge. This could mean that patients have been discharged from hospital too early.^12^

We have to consider several factors while modeling survival after heart attack. First, the length of in-hospital stay until death or discharge may be correlated with severity of the heart attack. Some hospitals may transfer patients early due to lack of skilled personnel or medical equipment required for treatment of heart attacks. Thus, we have to consider three durations modeled as competing risks—duration until in-hospital death, transfer and hospital discharge. Furthermore, the realization of all three durations might have an effect on post-discharge survival through both observed and unobserved factors. Despite the fact that our dataset includes relatively rich set of observed characteristics as well as type of heart attack (i.e. ST-elevated or non-ST-elevated), we are unable to assess the severity of heart attack based on more detailed clinical indicators.^13^ To take this potential dependence and unobserved factors into account, we estimate all four processes using a discrete mixture of unobserved heterogeneity following Heckman and Singer [17], where all unobserved components are allowed to be correlated with each other.

We model the in-hospital mortality rate *h*, discharge rate *s* and transfer rate *r* at duration since heart attack *t* (omitting the subscript for individuals) conditional on a vector of observed characteristics *x*, residence characteristics *w*, the local-area treatment intensity $T_{g}$ and unobserved characteristics $\upsilon$ as:

3 $$\begin{aligned} \theta _j (t \mid x, w, T_{g}, \upsilon _j) = \lambda _j(t) \exp (x'\beta _j+w'\gamma _j+\log (T_{g})\zeta _j+\upsilon _j) \quad {\text {for}} \quad j=h,s,r \end{aligned}$$

where $T_{g}$ is the local-area spending corresponding to the first hospital’s HSA in which a particular patient was hospitalised.^14^ Observed characteristics *x* are gender, age, comorbidities of the patient, whether the heart attack happened during a weekend and the quarter of the year.^15^ Residence characteristics *w* are median income, share of inhabitants with university education, and whether or not the residence is in a rural area, while $\upsilon$ represents a random effect capturing unobserved heterogeneity. Furthermore, $\lambda _j(t)$ represents individual duration dependence, which is flexibly modeled using a step function:

4 $$\begin{aligned} \lambda _j(t)=\exp \left( \sum _k \lambda _{j,k} I_k(t)\right) \end{aligned}$$

where $k(=1,\ldots ,K)$ is a subscript for day-intervals and $I_k(t_l)$ are time-varying dummy variables for subsequent day-intervals when the event (death) occurs. The intervals are defined for days 0–2, 3–4, 5–6, 7–10, 11–15 and more than 15 days. The conditional density function of durations until in-hospital death, hospital discharge or transfer to a different hospital is defined as:

5 $$\begin{aligned} \begin{aligned} f(t \mid x, w, T_{g}, \upsilon _h, \upsilon _s, \upsilon _r)&= \sum _j \theta _j (t \mid x, w, T_{g}, \upsilon _j) \\&\exp \left( -\int _0^{t} \sum _j \theta _j (u \mid x, w, T_{g}, \upsilon _j) \, du\right) \quad {\text {for}} \quad j=h,s,r \end{aligned} \end{aligned}$$

Note that *t* is equivalent to the hospital length of stay. The above formula represents the density function of the competing risk part of the model.

The post-discharge mortality rate at duration since hospital discharge $t_d$ conditional on observed characteristics *x*, residence characteristics *w*, post-discharge treatment intensity $T_g^P$ and unobserved characteristics $\nu$ is modeled similarly:

6 $$\begin{aligned} \theta _d (t_d \mid x, w, T_g^P, \nu ) = \lambda _d(t_d) \exp (x'\beta _d+w'\gamma _d+\log (T_g^P)\zeta _d+\nu ) \end{aligned}$$

Stepwise duration dependence $\lambda _d$ is defined as in Eq. 4 with intervals for days 0–2, 3–4, 5–8, 9–16, 17–30, 31–60, 61–180 and more than 180 days. The conditional density function of completed durations until death post-discharge can be written as:

7 $$\begin{aligned} \begin{aligned} f_d(t_d \mid x, w, T_g^P, \nu )&= \theta _d(t_d \mid x, w, T_g^P, \nu ) \\&\exp \left( -\int _0^{t_d} \theta _d(s \mid x, w, T_g^P, \nu ) \, ds\right) \end{aligned} \end{aligned}$$

The potential correlation between the unobserved components in the hazard rates for in-hospital mortality, hospital discharge, hospital transfer and post-discharge mortality is taken into account by specifying the joint density function for the duration of time until in-hospital death, transfer or discharge *t* and the duration of time until death after discharge $t_d$, conditional on *x*, *w* $T_g$ and $T_g^P$. We assume that the random effects $\upsilon _h$, $\upsilon _s$, $\upsilon _r$ and $\nu$ are specified following a discrete mixing distribution *G*, where each of the components has two points of support:

8 $$\begin{aligned} \begin{aligned} g(t, t_d \mid x, w, T_g, T_g^P)&= \int _{\upsilon _h} \int _{\upsilon _s} \int _{\upsilon _r} \int _\nu f(t \mid x, w, T_{g}, \upsilon _h, \upsilon _s, \upsilon _r) \\&f_d(t_d \mid x, w, T_g^P, \nu )\, dG(\upsilon _h, \upsilon _s, \upsilon _r, \nu ) \end{aligned} \end{aligned}$$

The full mixing distribution yields 16 possible combinations, each describing types of patients with different hazard rates of in-hospital mortality, hospital discharge, transfer from hospital and post-discharge mortality. The probabilities associated with 16 mass points of the joint distribution are defined as:

9 $$\begin{aligned} p_1&= \Pr (\nu _1, \upsilon _{s,1}, \upsilon _{h,1}, \upsilon _{r,1}),\quad p_2= \Pr (\nu _2, \upsilon _{s,1}, \upsilon _{h,1}, \upsilon _{r,1}) \nonumber \\ p_3&= \Pr (\nu _1, \upsilon _{s,2}, \upsilon _{h,1}, \upsilon _{r,1}),\quad p_4= \Pr (\nu _2, \upsilon _{s,2}, \upsilon _{h,1}, \upsilon _{r,1}) \nonumber \\ p_5&= \Pr (\nu _1, \upsilon _{s,1}, \upsilon _{h,2}, \upsilon _{r,1}),\quad p_6= \Pr (\nu _2, \upsilon _{s,1}, \upsilon _{h,2}, \upsilon _{r,1}) \nonumber \\ p_7&= \Pr (\nu _1, \upsilon _{s,2}, \upsilon _{h,2}, \upsilon _{r,1}),\quad p_8= \Pr (\nu _2, \upsilon _{s,2}, \upsilon _{h,2}, \upsilon _{r,1}) \nonumber \\ p_{10}&= \Pr (\nu _1, \upsilon _{s,1}, \upsilon _{h,1}, \upsilon _{r,2}), \quad p_{11}= \Pr (\nu _2, \upsilon _{s,1}, \upsilon _{h,1}, \upsilon _{r,2}) \nonumber \\ p_{11}&= \Pr (\nu _1, \upsilon _{s,2}, \upsilon _{h,1}, \upsilon _{r,2}), \quad p_{12}= \Pr (\nu _2, \upsilon _{s,2}, \upsilon _{h,1}, \upsilon _{r,2}) \nonumber \\ p_{13}&= \Pr (\nu _1, \upsilon _{s,1}, \upsilon _{h,2}, \upsilon _{r,2}), \quad p_{14}= \Pr (\nu _2, \upsilon _{s,1}, \upsilon _{h,2}, \upsilon _{r,2}) \nonumber \\ p_{15}&= \Pr (\nu _1, \upsilon _{s,2}, \upsilon _{h,2}, \upsilon _{r,2}), \quad p_{16}=\Pr (\nu _2, \upsilon _{s,2}, \upsilon _{h,2}, \upsilon _{r,2}) \end{aligned}$$

where $p_{n}$ is assumed to follow a multinomial logistic distribution:

10 $$\begin{aligned} p_n=\frac{\exp (\alpha _n)}{\sum _n \exp (\alpha _n)},\quad n=1,\ldots ,16 \end{aligned}$$

with $\alpha _{16}$ normalized to zero so $p_{16}=1-p_1-p_2-\cdots -p_{15}$. The parameters of the model are estimated using the method of maximum likelihood.

### Parameter estimates

Table 2 presents the main parameter estimates, i.e. the effects of health-care spending on mortality.^16^ Panel A presents estimates for in-hospital mortality based on the samples of locals and visitors, while panel B shows estimates for post-discharge mortality based on the samples of stayers and movers. Columns (1) and (3) present the parameter estimates if unobserved heterogeneity is ignored and the various hazard rates are estimated separately. Columns (2) and (4) shows the parameter estimates when the four hazard rates are jointly estimated taking potential correlation between the four unobserved heterogeneity terms into account.

Panel A shows that in-hospital spending has a significant negative effect on in-hospital mortality of locals. The differences between the parameter estimates in the two columns is small. So, taking unobserved heterogeneity into account is not very important. The same holds for the parameter estimates for visitors. The effects of in-hospital spending on in-hospital mortality are also very similar for locals and visitors which suggests that selectivity or effects being contaminated by other differences between HSAs is not very important either.

From panel B it is clear that post-discharge spending has a significant negative effect on post-discharge mortality for both stayers and movers. The magnitude of the effect is larger for movers than it is for stayers although the parameter estimates for movers are somewhat imprecisely estimated.^17^

**Table 2:** Health outcomes for heart attacks—duration models

Mortality rate	(1)	(2)	(3)	(4)
*Panel A: In-hospital mortality*				
	Locals		Visitors	
In-hospital spending ($\log T_g$)	− 0.753	− 0.767	− 0.675	− 0.719
	(5.5)***	(3.7)***	(2.4)**	(1.9)*
Average mortality	0.046		0.054	
Observations	41,127		3521	
*Panel B: Post-discharge mortality*				
	Stayers		Movers	
Post-discharge spending ($\log T_g^P$)	− 0.289	− 0.280	− 0.924	− 0.971
	(2.0)**	(3.1)**	(1.9)*	(1.9)*
Average mortality	0.219		0.175	
Observations	43,510		1138	
Unobserved heterogeneity	No	Yes	No	Yes

All estimates contain personal characteristics and residence characteristics. The full set of estimates for all equations of the main model is presented in Appendix Tables 8, 9, 10 and 11. Robust absolute *t* statistics clustered at the HSA level in parentheses

*$$p<0.10$$, **$$p<0.05$$, ***$$p<0.001$$

The full set of parameter estimates is presented in Appendix Tables 8, 9, 10 and 11. Table 9 shows that age has a positive effect on mortality. Older patients stay in hospital longer than younger patients. Males are as likely to die in hospital as females while they are more likely to die after hospital discharge. Many comorbidities increase mortality rates in and out of hospital and they increase hospital stay. In-hospital mortality is highest in the first duration interval and approximately constant after that. Post-discharge mortality rates exhibit negative duration dependence; hospital discharge rates have positive duration dependence. While we model a discrete distribution of unobserved heterogeneity with 16 points of support in practice only a few points of support are identified. Conditional on observed characteristics and duration dependence there are three to five types of patients who differ in unobserved terms from each other. Often the unobserved heterogeneity is highly correlated between in-hospital mortality and post-discharge mortality suggesting that unobserved health status or unobserved severity of the heart attack are important.

### Sensitivity analysis

To investigate the robustness of our main findings we perform some sensitivity analysis on the group of movers and visitors, focusing on potential heterogeneity by gender, age and comorbidities.^18^ The results are shown in Table 3. Columns (1) and (3) report the parameter estimates of post-discharge spending, while columns (2) and (4) report the estimates for in-hospital costs. The results are based on our baseline estimate presented in column (4) of Table 2. Overall, the results are in line with the baseline estimates. All coefficients have negative sign, although not all parameters are precisely estimated. The effect of post-discharge spending on post-discharge mortality seems to be higher for those older than age 65. There is some evidence of gender-specific heterogeneity, since the effect of in-hospital spending is not different from zero for males. Finally, we also investigate whether the results for post-discharge mortality are not driven by observing certain patients for only a short period of time. In order to do so, we extend the observation period of movers sample up to the end of year 2021 and define the outcome variable as 2-year mortality. Durations beyond 730 days are considered as right-censored at the 730th day. This way all patients in the sample are observed for at least 2 years since discharge (our main sample considers all AMI-related hospitalisations up to December 31, 2019). The estimated coefficient of post-discharge mortality is even larger in magnitude and statistically significant at the 5% level (not reported).

**Table 3:** Health outcomes for heart attacks—sensitivity analysis

	Movers	Visitors	Movers	Visitors
	Mortality	In-hospital mortality	Mortality	In-hospital mortality
	(1)	(2)	(3)	(4)
*Panel A: By age*				
	Age $<65$		Age $\ge 65$	
Spending log($T^P_g$), log($T_g$)	− 0.854	− 0.412	− 0.982	− 0.694
	(1.7)*	(1.1)	(1.9)*	(1.5)
Observations	1138	1641	1138	1880
*Panel B: By gender*				
	Males		Females	
Spending log($T^P_g$), log($T_g$)	− 0.924	− 0.656	− 0.921	− 0.829
	(1.8)*	(1.3)	(1.8)*	(2.3)**
Observations	1138	2250	1138	1271
*Panel C: By comorbidity status*				
	Charlson index $=0$		Charlson index $\ge 1$	
Spending log($T^P_g$), log($T_g$)	− 0.923	− 0.848	− 0.936	− 0.624
	(1.8)*	(1.8)*	(1.9)*	(1.3)
Observations	1138	1710	1138	1811

All estimates include full set of controls and UH. Due to low number of events for certain categorical variables in split samples, estimates for movers are based on a pooled sample with interaction terms for respective groups. Robust absolute *t* statistics clustered at the HSA level in parentheses

*$$p<0.10$$, **$$p<0.05$$, ***$$p<0.001$$

In additional sensitivity analysis, we estimated separate models for in-hospital spending of urban and rural HSAs, based on average population density of districts comprising HSAs. Urban HSAs are defined as having more than 150 inhabitants per square kilometer. The results are robust, although the effects of in-hospital spending on mortality are somewhat less precisely estimated for the urban HSAs. This is likely related to the fact that the urban HSAs are mostly concentrated within the top quartile of the spending distribution, where the spending is already high. Thus, the differences in spending at the top of the distribution may have less of an effect on mortality.

### Magnitude of the effects

To indicate the magnitude of various effects, we performed simulations based on parameter estimates for movers and visitors from our baseline specification in Table 2. The results are summarized in Table 4. The simulations of the effects are based on a reference patient, defined as a male individual with the sample median age of 67 years, hospitalized for the sample mean of five days and having two common comorbidities (diabetes and cardiovascular disease). The predicted mortality is then simulated over the spending distribution. To show how the predicted mortality changes when the spending is fixed, the exercise is repeated across the age distribution. The simulation of the age effects is based on a male individual with the same comorbidities, hospitalised or residing in the median spending HSA. Finally, the simulation of comorbidity effects is again based on a median-aged male, with median HSA-spending. More details about the simulations are provided in “Magnitude of effects” of appendix.

**Table 4:** Effects of spending and age on mortality

Spending effects			Age effects		
Spending percentile		Predicted mortality	Age percentile		Predicted mortality
*Panel A: In-hospital mortality at 5 days*					
1%	(1141€)	15.8	99%	(91 years)	27.4
25%	(1339€)	15.0	75%	(76 years)	18.9
50%	(1436€)	16.0	50%	(67 years)	13.9
75%	(2697€)	12.7	25%	(59 years)	10.6
99%	(3041€)	9.0	1%	(37 years)	4.7
*Panel B: Post-discharge mortality at 1 year*					
1%	(192€)	19.0	99%	(91 years)	50.8
25%	(250€)	15.5	75%	(76 years)	24.0
50%	(282€)	14.0	50%	(67 years)	14.0
75%	(301€)	13.2	59%	(59 years)	8.3
99%	(367€)	11.0	1%	(37 years)	2.0

It is clear that being hospitalized with AMI in the lowest spending HSA coincides with a substantial higher mortality than being hospitalized in a higher-spending HSA. An increase of in-hospital spending from the first to the third quartile of spending distribution reduces mortality for a median male^19^ patient by 2.3 percentage points, while an increase of spending from the 1st to 99th percentile is associated with an decrease of mortality of almost seven percentage points. For a comparison, Doyle [7] reports a difference of 2.8 percentage points in mortality between the first and third quartile of spending distribution. There are also significant differences in mortality by age. A patient aged 59 years hospitalized in a median-spending HSA has a predicted mortality of almost 11%, whereas the same patient aged 76 years has a mortality expectation that is about eight percentage points higher.

The effects of post-discharge spending on mortality at 1 year since discharge are even more pronounced at the tails of the distribution when compared to the effects of in-hospital spending. The interquartile range of spending is equal to 51€ and is associated with a similar decrease in mortality as with in-hospital spending. As with in-hospital costs, age has substantial effect on mortality if the spending is assumed at the median HSA. A 76-years-old patient has a mortality rate that is nearly 16 percentage points higher compared to the same patient aged 59.

## Mechanisms

The finding that higher health-care spending is associated with lower mortality is interesting. However, this does not rule out the possibility that lower mortality does not solely depend on higher health-care spending but also to some regions for example having better physicians. Nevertheless, we can explore potential mechanisms in more detail. For the in-hospital spending we do not have detailed information about procedures used but for post-discharge spending we have disaggregate information. This is helpful in understanding whether there is a relationship between higher spending and the use of particular health-care procedures.^20^ To investigate whether this is the case, we select the top 10 most frequent used procedures performed for patients following discharge from acute care hospitals, conditional on surviving at least 6 months after the AMI. The counts for the procedures at the patient level are then estimated using a zero-inflated Poisson regression, with all explanatory variables as used in the main specifications presented in previous sections. The results are summarized in Table 5.

The most frequent post-discharge procedure among AMI patients is a standard physical examination performed by a general practitioner (GP) doctor. GPs in Slovakia issue referrals and serve as gatekeepers for other primary care specialists. The estimated coefficient on treatment intensity for these procedures is positive, suggesting that higher-spending HSAs have a higher incidence of GP encounters for AMI patients. The three following procedures are specific blood tests. Electrolyte imbalances in AMI patients are not uncommon, while maintenance of adequate serum levels is critical to prevent adverse events such as ventricular arrythmias [20]. Activated partial thromboplastin time (aPTT) and prothrombin time (PT) measure function of blood clotting. Heart attacks are often caused by a formation of blood clots blocking arteries delivering blood to the heart. Due to this, patients after AMI are often prescribed anti-coagulants. Granger et al. [21] for example find an association between increased aPTT and risk of reinfarction. Cardiac markers such as troponin and creatine kinase (CK) are indicators of muscle damage, i.e. are often elevated after AMI. However, the estimated coefficients suggest that HSAs with higher spending perform significantly less of the three laboratory investigations. This may be explained by the fact that continuous monitoring of blood levels of troponin and CK is unlikely to be effective, since both markers are elevated only shortly after heart attack.

Electrocardiography (ECG) is a non-invasive procedure capable of detecting subtle changes in electrical activity of the heart, which is often indicative of various cardiac abnormalities. Studies such as Gill et al. [22] note that ambulatory ECG monitoring of patients after AMI is important to determine the risk of a subsequent coronary event. Our estimates suggest that higher-spending HSAs perform significantly more ECGs than lower spending HSAs. Although not precisely estimated, the use of other non-invasive imaging procedures such as color flow mapping (CFM), pulse-wave (PW) and continuous-wave (CW) Doppler echocardiography (ultrasound imaging of heart) is pointing in the same direction.

Coronary angiography is a medical imaging technique used to visualize blood vessels. However, according to medical guidelines, its routine use in stable patients is discouraged [23]. Thus, from a cost-effectiveness perspective, it is perhaps not surprising that higher-spending regions are performing significantly smaller quantity of these procedures. Finally, patients recovering from AMI often benefit from tailored rehabilitation programs, which include lighter forms of exercise. While there seems to be no apparent difference between low- and high-spending regions in use of exercise therapy solely, there seems to be an increased use of a more complex cardiac rehabilitation.^21^

From all this, we conclude that the reduction in post-discharge mortality in higher-spending regions is likely related to better monitoring of patients, as evidenced by an increased use of ECG, as well as more frequent visits to GP. The use of cardiac rehabilitation programs also appears to be important.

**Table 5:** Coefficients on HSA post-discharge treatment intensity; top 10 post-discharge procedures among AMI patients

Procedure codes	Procedure	Count	Coefficient	*t*-statistic
4, 8, 60, 62, 63	Physical examinations	44,938	0.198	(1.1)
3704, 3705, 3706	Electrolytes (Sodium/potassium)	33,188	− 0.760	(3.0)**
3842, 3852	Blood clotting (PT/aPTT)	13,259	− 0.667	(1.5)
4485, 3696	Cardiac markers (troponin/CK)	12,504	− 0.870	(3.0)**
15c, 5702, 5708	Electrocardiography (ECG)	6729	1.486	(2.3)**
5744	CFM echocardiography	3310	0.332	(0.3)
5745	PW/CW Doppler echocardiography	3244	1.010	(0.6)
512, 513, 514	Exercise therapy	2184	0.120	(0.2)
5110, 5120, 5121, 5122, 5206, 5612b	Coronary angiography	2173	− 1.584	(3.3)**
87a	Cardiac rehabilitation	877	1.835	(1.5)

All models estimated with full set of controls as in the main specifications using zero-inflated Poisson regression, where zero counts are modeled as logit. Robust absolute *t* statistics clustered at the HSA level in parentheses

*$$p<0.10$$, **$$p<0.05$$, ***$$p<0.001$$

## Conclusions

This paper uses administrative data to investigate the effects of health-care spending on mortality rates of patients who experienced a heart attack. We distinguish between in-hospital mortality which we relate to in-hospital spending and post-discharge mortality which we relate to post-discharge health-care spending.

In the analysis of in-hospital mortality we distinguish between two types of patients, i.e., locals and visitors. Locals use hospital services in their region; visitors are hospitalized in regions different from their region of residence. The distinction between the two types of patients helps us to identify the causal effect of health-care costs on mortality. It is possible that regional variations in health-care expenditures are related to the health status of the population; i.e. regions with many unhealthy people may spend more on health care. Regions with many unhealthy people may also have a higher mortality. This induces selectivity, i.e., a cross-regional positive association between health-care spending and mortality. Therefore, it may be that the negative effect of health-care spending on mortality is underestimated or even reversed. For visitors this contamination of negative causal effects and positive association is not present. This means that for visitors relating their in-hospital mortality after heart attack to in-hospital expenditures gives a clean estimate of the treatment effect. We find for both locals and visitors a negative effect of in-hospital health-care costs on in-hospital mortality. These effects are of the same magnitude which suggests that selectivity is not an important issue and there is a negative causal effect of in-hospital health-care expenditures on in-hospital mortality.

For post-discharge expenditures and post-discharge mortality a comparison between locals and visitors is not helpful to get idea about causal effects since also visitors are likely to be treated in their region of residence. A plausibly exogenous group to analyze the relationship between post-discharge mortality and spending consists of patients who migrate between regions before experiencing a heart attack. For this sample, we also find significantly negative effects of higher spending on mortality.

All in all, we interpret the negative effects of higher health-care spending on mortality as causal effects. Regional variation in mortality is to a large extent related to regional variation in health-care expenses. The regional variation in health-care spending is related to regional variation in wealth and population density. From this, we conclude that from a health perspective it is better to have a heart attack in a wealthy and densely populated region than to have a heart attack in a poor and sparsely populated region.

## Data Availability

The data that support the findings of this study are available on request. The data are not publicly available due to privacy or ethical restrictions.

## Appendix 1

### Sensitivity analysis

As an additional sensitivity analysis we replicate Doyle [7], who uses linear probability models (LPM) explaining in-hospital mortality for patients and visitors estimating the parameters of the following equation:

11 $$\begin{aligned} M_i = \alpha + \log (T_{gi})\zeta + x_i'\beta + w_{ci}'\gamma + \epsilon _i \end{aligned}$$

where $M_i$ represent a binary indicator denoting whether patient *i* died in hospital, $T_{gi}$ is the in-hospital spending in HSA region *g*, *x* is a vector of observed characteristics of the patient and treatment characteristics, *w* denotes vector of characteristics of residence *c* in which the patient was hospitalized, and $\epsilon _i$ represents an error term. Note that *x* and *w* include explanatory variables as defined previously in our main specifications.

Panel A of Table 6 presents the parameter estimates related to in-hospital health-care costs. Columns (1) and (2) report estimates for locals (without and with residence characteristics), while columns (3) and (4) report estimates for visitors (without and with residence characteristics). All parameter estimates are significantly negative—the higher the in-hospital costs the lower in-hospital mortality. Contrary to Doyle, we also find a significant negative effect of local-area treatment intensity for local patients and not just for visitors. Interestingly, the magnitude of the parameter estimates is similar for locals and visitors. This suggests that selectivity in regional treatment is not an issue.

In panel B, estimates of a LPM model for post-discharge mortality are presented, where $M_i$ now denotes a binary variable equal to 1 if patient died after discharge from hospital within 2 years, restricting the sample to patients for whom we have information for at least 2 years since discharge. For stayers the effect of post-discharge health-care spending is negative and statistically significant. For movers the coefficients are also negative, but imprecisely estimated. This also implies that the parameter estimates of stayers and movers are not significantly different from each other. This again confirms that regional selectivity is not an issue.

Table 7 shows the full set of parameter estimates related to those presented in Table 6. From these it appears that many of the mortality effects are similar in-hospital and post-discharge. Mortality increases with age, while post-discharge mortality of males is higher and about the same as for females in hospital. Mortality increases with the length of hospital stay but this is most likely reverse causality, i.e., those who stay longer in hospital are more likely to die. Those with median income in the highest quartile have a higher post-discharge mortality, similarly to those living in rural areas. Nearly all comorbid conditions significantly increase mortality after discharge from hospital.

**Table 6:** Health outcomes for heart attacks—linear probability models

Dependent variable: mortality rate	(1)	(2)	(3)	(4)
*Panel A: In-hospital mortality*				
	Locals		Visitors	
In-hospital spending ($\log T_g$)	− 0.035	− 0.043	− 0.045	− 0.047
	(2.2)**	(3.7)***	(2.2)**	(2.4)**
Mean of dependent variable	0.046		0.054	
Observations	41,127		3521	
*Panel B: 2-year post-discharge mortality*				
	Stayers		Movers	
Post-discharge spending ($\log T_g^P$)	− 0.053	− 0.059	− 0.032	− 0.124
	(2.2)**	(2.5)**	(0.4)	(1.2)
Mean of dependent variable	0.195		0.135	
Observations	25,361		652	
Residence characteristics	No	Yes	No	Yes

All estimates include personal characteristics. Robust absolute *t* statistics clustered at the HSA level in parentheses

*$$p<0.10$$, **$$p<0.05$$, ***$$p<0.001$$

**Table 7:** Parameter estimates LPM models

	Locals		Stayers		Visitors		Movers	
	Mortality		In-hospital mortality		Mortality		In-hospital mortality	
	(1)		(2)		(3)		(4)	
Health-care spending								
In-hospital ($\log T_g$)	− 0.04	(3.7)***			− 0.05	(2.4)**		
Post-discharge ($\log T^P_g$)			− 0.06	(2.5)**			− 0.12	(1.2)
Personal characteristics								
Age	0.00	(8.3)***	0.01	(30.5)***	0.00	(5.6)***	0.01	(7.1)***
Male	− 0.00	(1.0)	0.00	(0.6)	− 0.00	(0.2)	− 0.03	(1.2)
AMI of inferior wall (I21.1)	− 0.01	(3.6)**	− 0.03	(3.8)***	− 0.00	(0.2)	0.01	(0.2)
AMI of other sites (I21.2)	− 0.01	(2.6)**	0.02	(1.3)	− 0.01	(0.4)	− 0.08	(1.8)*
AMI of unspecified site (I21.3)	0.03	(1.4)	0.04	(2.2)**	0.01	(0.7)	0.03	(0.5)
Non-ST elevation AMI (I21.4)	− 0.04	(5.7)***	− 0.02	(3.4)**	− 0.06	(6.4)***	0.02	(0.5)
AMI, unspecified (I21.9)	0.02	(1.7)	0.01	(1.5)	0.04	(2.8)**	0.03	(0.8)
Length of stay	− 0.01	(8.4)***	0.01	(9.3)***	− 0.01	(5.5)***	0.00	(1.5)
Residence characteristics								
Median income 950€-1010€	− 0.00	(0.5)	− 0.00	(0.0)	0.01	(0.9)	0.07	(1.3)
Median income 1010€-1105€	0.00	(0.1)	− 0.00	(0.4)	0.04	(2.8)**	0.10	(2.5)**
Median income >1105€	0.01	(0.4)	0.02	(2.3)**	0.05	(3.8)***	0.08	(1.5)
University	0.00	(1.5)	− 0.00	(3.0)**	− 0.00	(1.1)	0.00	(0.5)
Rural area	0.00	(0.7)	0.00	(0.4)	− 0.01	(1.2)	0.07	(1.5)
Timing								
Weekend	0.00	(1.7)	0.02	(3.4)**	− 0.01	(1.4)	0.01	(0.2)
2nd quarter	− 0.01	(2.2)**	− 0.00	(0.4)	− 0.02	(1.5)	0.08	(1.9)*
3rd quarter	− 0.00	(1.1)	0.00	(0.6)	− 0.00	(0.4)	0.04	(1.3)
4th quarter	− 0.00	(1.2)	0.01	(0.8)	− 0.00	(0.3)	0.08	(2.6)**
Comorbidities								
Recent AMI	− 0.02	(4.5)***	0.02	(2.4)**	− 0.03	(2.6)**	− 0.00	(0.0)
Cardiovascular disease	0.04	(3.7)***	0.21	(13.9)***	0.04	(2.2)**	0.17	(2.5)**
Cerebrovascular disease	0.03	(3.1)**	0.14	(6.4)***	0.02	(0.9)	0.11	(1.0)
Gastrointestinal disease	0.02	(1.8)*	0.07	(4.0)***	0.06	(1.4)	− 0.05	(0.3)
Diabetes	0.01	(1.8)*	0.06	(9.3)***	0.01	(0.7)	− 0.06	(1.6)
Respiratory disease	0.02	(3.4)**	0.06	(4.7)***	0.02	(1.3)	− 0.01	(0.1)
Cancer/AIDS	0.01	(1.9)*	0.13	(9.1)***	0.03	(1.2)	0.17	(1.3)
Other comorbidities	0.05	(4.2)***	0.19	(10.9)***	0.02	(1.0)	0.26	(2.5)**
Constant	0.22	(2.4)**	− 0.11	(0.9)	0.30	(1.9)*	0.30	(0.5)
Observations	41,127		25,361		3521		621	

Robust absolute *t* statistics clustered at the HSA level in parentheses

*$$p<0.10$$, **$$p<0.05$$, ***$$p<0.001$$

## Appendix 2

### Additional results and tables

See Tables 8, 9, 10, 11, 12 and 13.

**Table 8:** Parameter estimates separate processes—in-hospital spending

	Locals								Visitors							
	Mortality		Hospital discharge		In-hospital mortality		Hospital transfer		Mortality		Hospital discharge		In-hospital mortality		Hospital transfer	
	(1)		(2)		(3)		(4)		(5)		(6)		(7)		(8)	
Health-care spending																
In-hospital ($\log T_g$)	− 0.12	(1.9)*	0.21	(2.4)**	− 0.75	(5.5)***	− 0.31	(1.5)	− 0.06	(0.4)	0.24	(2.4)**	− 0.68	(2.4)**	− 0.55	(2.5)**
Personal characteristics																
Age	0.07	(45.9)***	− 0.01	(16.4)***	0.06	(16.3)***	− 0.02	(10.6)***	0.06	(13.7)***	− 0.01	(12.0)***	0.04	(5.4)***	− 0.02	(9.2)***
Male	0.09	(3.9)***	0.02	(1.7)*	0.01	(0.2)	0.12	(6.8)***	0.18	(1.9)*	0.01	(0.5)	0.07	(0.4)	0.02	(0.2)
AMI of inferior wall (I21.1)	− 0.20	(4.8)***	0.10	(6.0)***	− 0.20	(2.9)**	0.08	(2.0)**	− 0.20	(1.5)	0.08	(2.0)**	0.20	(0.9)	0.13	(1.7)*
AMI of other sites (I21.2)	− 0.02	(0.2)	0.00	(0.1)	− 0.28	(2.9)**	− 0.02	(0.3)	0.19	(1.1)	0.03	(0.3)	− 0.05	(0.1)	0.31	(1.3)
AMI of unspecified site (I21.3)	0.23	(3.2)**	− 0.15	(4.4)***	0.30	(2.5)**	− 0.62	(7.1)***	− 0.01	(0.0)	− 0.23	(2.4)**	0.03	(0.1)	− 0.35	(1.4)
Non-ST elevation AMI (I21.4)	− 0.09	(1.9)*	− 0.11	(5.7)***	− 1.19	(16.8)***	− 0.25	(3.8)***	− 0.30	(2.6)**	0.02	(0.4)	− 1.41	(5.1)***	0.07	(0.4)
AMI, unspecified (I21.9)	0.07	(1.7)*	− 0.24	(6.2)***	− 0.02	(0.2)	− 0.23	(2.2)**	− 0.08	(0.7)	− 0.13	(2.4)**	0.35	(1.9)*	− 0.08	(0.6)
Length of stay	0.02	(8.6)***							0.02	(2.7)**						
Residence characteristics																
Median income 950€-1010€	− 0.05	(1.0)	0.01	(0.3)	− 0.06	(0.5)	0.13	(1.6)	− 0.01	(0.1)	− 0.08	(1.1)	0.14	(0.7)	0.22	(1.3)
Median income 1010€-1105€	− 0.07	(1.4)	− 0.01	(0.1)	0.01	(0.1)	0.07	(0.6)	0.04	(0.3)	− 0.17	(2.3)**	0.40	(2.0)*	− 0.00	(0.0)
Median income >1105€	0.03	(0.5)	0.01	(0.2)	0.08	(0.5)	0.40	(2.8)**	0.17	(1.3)	− 0.17	(1.4)	0.81	(2.6)**	0.06	(0.3)
University educated	− 0.01	(2.6)**	− 0.01	(3.8)***	0.01	(1.6)	− 0.03	(2.9)**	− 0.02	(2.8)**	0.00	(0.5)	− 0.02	(0.6)	− 0.01	(1.0)
Rural area	0.00	(0.1)	− 0.05	(2.8)**	0.01	(0.1)	− 0.06	(1.2)	0.01	(0.1)	− 0.02	(0.4)	− 0.21	(1.0)	− 0.16	(0.8)
Timing																
Weekend	0.06	(2.1)**	0.06	(7.7)***	0.12	(2.2)**	0.17	(8.0)***	0.10	(0.9)	0.11	(2.4)**	− 0.20	(1.0)	0.15	(1.7)*
2nd quarter	− 0.01	(0.3)	− 0.02	(1.6)	− 0.15	(2.8)**	− 0.06	(2.2)**	0.12	(1.3)	0.00	(0.1)	− 0.35	(1.3)	− 0.08	(0.8)
3rd quarter	0.04	(1.1)	− 0.02	(1.2)	− 0.07	(1.2)	− 0.04	(2.0)**	− 0.02	(0.2)	− 0.04	(1.1)	− 0.11	(0.8)	− 0.14	(1.4)
4th quarter	0.03	(1.1)	− 0.01	(0.4)	− 0.08	(1.2)	− 0.01	(0.3)	− 0.03	(0.4)	− 0.01	(0.2)	0.01	(0.1)	− 0.01	(0.1)
Comorbidities																
Recent AMI	0.10	(3.8)***	− 0.00	(0.1)	− 0.48	(6.2)***	− 0.89	(16.7)***	0.13	(1.5)	− 0.03	(0.6)	− 0.71	(3.0)**	− 1.00	(6.8)***
Cardiovascular disease	0.69	(19.7)***	− 0.14	(9.5)***	0.44	(4.1)***	− 0.23	(3.8)***	0.53	(5.0)***	− 0.18	(3.3)***	0.53	(2.3)**	− 0.51	(3.4)***
Cerebrovascular disease	0.42	(6.7)***	− 0.15	(4.6)***	0.37	(3.1)**	− 0.22	(3.4)***	0.66	(3.4)***	− 0.07	(0.9)	0.35	(1.3)	− 0.60	(2.0)**
Gastrointestinal disease	0.25	(3.8)***	− 0.05	(1.5)	0.27	(2.5)**	− 0.12	(1.6)	0.13	(0.6)	− 0.25	(1.8)*	0.37	(0.9)	− 0.88	(2.3)**
Diabetes	0.42	(15.3)***	− 0.05	(4.4)***	0.18	(2.8)**	− 0.00	(0.1)	0.33	(3.6)***	− 0.07	(2.2)**	0.10	(0.5)	0.05	(0.7)
Respiratory disease	0.28	(6.6)***	− 0.03	(1.9)*	0.29	(4.1)***	− 0.07	(2.0)**	0.36	(2.5)**	− 0.05	(1.2)	0.37	(1.8)*	− 0.23	(1.4)
Cancer/AIDS	0.49	(11.7)***	− 0.04	(2.1)**	0.20	(2.8)**	− 0.07	(1.3)	1.09	(9.1)***	− 0.18	(2.7)**	0.35	(1.3)	− 0.39	(1.8)*
Other comorbidities	0.63	(12.5)***	− 0.17	(4.6)***	0.47	(4.5)***	− 0.27	(3.9)***	0.70	(3.7)***	− 0.14	(2.0)**	0.04	(0.1)	− 0.16	(0.7)
Duration dependence																
Constant	− 10.25	(20.2)***	− 2.12	(3.3)***	− 0.85	(6.3)***	1.21	(0.8)	− 10.58	(7.9)***	− 2.63	(3.5)***	− 1.38	(0.6)	3.16	(2.2)**
Duration 2	0.27	(2.8)**	0.23	(5.0)***	− 2.43	(2.5)**	− 0.23	(3.9)***	0.36	(1.3)	0.29	(5.4)***	− 1.10	(4.9)***	− 0.11	(0.7)
Duration 3	0.04	(0.4)	0.40	(5.9)***	− 1.11	(20.2)***	− 0.23	(2.0)**	0.46	(1.4)	0.52	(7.7)***	− 1.23	(4.4)***	− 0.10	(0.7)
Duration 4	− 0.26	(3.0)**	0.33	(7.9)***	− 1.12	(13.7)***	− 0.29	(2.5)**	− 0.01	(0.0)	0.39	(4.9)***	− 1.14	(4.2)***	− 0.12	(0.7)
Duration 5	− 0.78	(8.8)***	0.08	(1.7)*	− 1.07	(13.4)***	− 0.90	(6.2)***	− 0.40	(1.2)	0.15	(1.8)*	− 1.22	(3.2)**	− 0.61	(2.7)**
Duration 6	− 1.20	(15.1)***	− 0.29	(4.4)***	− 1.05	(8.0)***	− 2.06	(13.4)***	− 0.68	(2.2)**	0.08	(0.6)	− 0.55	(1.5)	− 1.43	(3.4)***
Duration 7	− 1.80	(21.4)***							− 1.39	(4.9)***						
Duration 8	− 2.30	(30.1)***							− 1.98	(6.5)***						
− Log likelihood	217,285.9								18,672.6							
Observations	41,127								3521							

Robust absolute *t* statistics clustered at the HSA level in parentheses

*$$p<0.10$$, **$$p<0.05$$, ***$$p<0.001$$

**Table 9:** Parameter estimates correlated model—in-hospital spending

	Locals								Visitors							
	Mortality		Hospital discharge		In-hospital mortality		Hospital transfer		Mortality		Hospital discharge		In-hospital mortality		Hospital transfer	
	(1)		(2)		(3)		(4)		(5)		(6)		(7)		(8)	
Health-care spending																
In-hospital ($\log T_g$)	− 0.14	(1.9)*	0.29	(2.0)**	− 0.77	(3.7)***	− 0.09	(0.4)	− 0.10	(0.5)	0.31	(2.1)**	− 0.72	(1.9)*	− 0.34	(1.1)
Personal Characteristics																
Age	0.07	(31.4)***	− 0.01	(7.4)***	0.06	(12.3)***	− 0.02	(4.3)***	0.07	(8.7)***	− 0.02	(7.6)***	0.04	(6.1)***	− 0.03	(5.4)***
Male	0.10	(4.4)***	0.01	(0.9)	0.01	(0.2)	0.10	(2.8)**	0.21	(2.1)**	− 0.00	(0.1)	0.01	(0.1)	− 0.08	(0.8)
AMI of inferior wall (I21.1)	− 0.22	(4.1)***	0.11	(4.2)***	− 0.20	(2.6)**	0.12	(4.4)***	− 0.29	(2.3)**	0.08	(1.3)	0.19	(0.8)	0.15	(1.2)
AMI of other sites (I21.2)	− 0.04	(0.5)	− 0.00	(0.0)	− 0.29	(2.3)**	− 0.04	(0.3)	0.16	(1.2)	0.02	(0.2)	− 0.02	(0.0)	0.36	(1.4)
AMI of unspecified site (I21.3)	0.24	(3.2)**	− 0.22	(1.9)*	0.33	(1.5)	− 0.82	(3.8)***	0.01	(0.1)	− 0.26	(2.8)**	− 0.15	(0.3)	− 0.45	(1.2)
Non-ST elevation AMI (I21.4)	− 0.13	(3.4)***	− 0.15	(3.0)**	− 1.21	(11.6)***	− 0.37	(2.3)**	− 0.51	(4.0)***	− 0.02	(0.4)	− 1.56	(6.3)***	− 0.05	(0.2)
AMI, unspecified (I21.9)	0.07	(1.5)	− 0.30	(2.8)**	0.02	(0.1)	− 0.43	(1.8)*	− 0.12	(1.1)	− 0.18	(2.1)**	0.40	(2.0)**	− 0.29	(1.1)
Length of stay	0.02	(4.1)***							0.02	(2.2)**						
Residence characteristics																
Median income 950€-1010€	− 0.04	(0.7)	− 0.01	(0.1)	− 0.04	(0.3)	0.08	(1.0)	− 0.01	(0.1)	− 0.08	(1.2)	0.07	(0.2)	0.24	(0.7)
Median income 1010€-1105€	− 0.07	(1.3)	− 0.02	(0.3)	− 0.00	(0.0)	0.03	(0.2)	0.09	(0.4)	− 0.21	(1.9)*	0.55	(1.6)	− 0.11	(0.4)
Median income >1105€	0.04	(0.5)	− 0.01	(0.1)	0.10	(0.4)	0.33	(1.3)	0.27	(1.1)	− 0.23	(1.9)*	0.99	(1.8)*	− 0.09	(0.3)
University educated	− 0.01	(2.6)**	− 0.01	(1.8)*	0.01	(1.4)	− 0.03	(3.0)**	− 0.03	(2.2)**	0.00	(0.4)	− 0.03	(0.8)	− 0.02	(0.8)
Rural area	− 0.00	(0.0)	− 0.07	(2.5)**	0.01	(0.2)	− 0.13	(3.8)***	0.02	(0.1)	− 0.03	(0.7)	− 0.28	(1.1)	− 0.20	(1.0)
Timing																
Weekend	0.08	(3.0)**	0.05	(2.3)**	0.16	(3.1)**	0.13	(2.0)**	0.12	(1.6)	0.06	(1.6)	− 0.17	(0.7)	− 0.01	(0.1)
2nd quarter	− 0.01	(0.5)	− 0.03	(1.9)*	− 0.17	(2.4)**	− 0.08	(2.7)**	0.05	(0.5)	0.01	(0.1)	− 0.41	(1.5)	− 0.07	(0.6)
3rd quarter	0.04	(1.3)	− 0.02	(1.8)*	− 0.07	(1.0)	− 0.07	(2.5)**	− 0.05	(0.4)	− 0.08	(1.4)	− 0.10	(0.6)	− 0.23	(2.0)**
4th quarter	0.03	(1.0)	− 0.02	(0.8)	− 0.09	(1.3)	− 0.03	(0.8)	− 0.06	(0.5)	− 0.00	(0.1)	− 0.07	(0.3)	0.02	(0.1)
Comorbidities																
Recent AMI	0.10	(3.3)**	0.00	(0.1)	− 0.49	(5.3)***	− 0.87	(5.4)***	0.12	(1.1)	− 0.05	(1.0)	− 0.75	(3.0)**	− 1.04	(3.4)***
Cardiovascular disease	0.75	(19.9)***	− 0.14	(4.8)***	0.49	(3.7)***	− 0.21	(2.2)**	0.86	(4.0)***	− 0.23	(1.7)*	0.78	(2.3)**	− 0.65	(1.1)
Cerebrovascular disease	0.46	(6.0)***	− 0.17	(3.8)***	0.40	(2.9)**	− 0.29	(2.6)**	0.81	(3.5)***	− 0.07	(1.0)	0.22	(0.6)	− 0.54	(1.8)*
Gastrointestinal disease	0.27	(3.9)***	− 0.06	(1.4)	0.30	(2.4)**	− 0.14	(1.8)*	− 0.08	(0.3)	− 0.30	(1.9)*	0.55	(1.4)	− 1.08	(2.4)**
Diabetes	0.45	(18.7)***	− 0.06	(3.6)***	0.20	(2.5)**	− 0.02	(0.4)	0.33	(3.7)***	− 0.06	(1.2)	0.08	(0.4)	0.06	(0.5)
Respiratory disease	0.30	(6.0)***	− 0.03	(1.0)	0.30	(5.6)***	− 0.05	(0.7)	0.49	(2.3)**	− 0.12	(2.8)**	0.52	(1.9)*	− 0.44	(2.6)**
Cancer/AIDS	0.52	(11.3)***	− 0.03	(0.9)	0.17	(2.3)**	− 0.02	(0.2)	1.48	(6.5)***	− 0.22	(2.1)**	0.65	(1.5)	− 0.45	(1.8)*
Other comorbidities	0.69	(12.4)***	− 0.19	(3.6)***	0.52	(4.4)***	− 0.34	(3.2)**	0.88	(4.6)***	− 0.16	(1.6)	0.17	(0.5)	− 0.25	(0.7)
Duration dependence																
Duration 2	0.27	(2.7)**	0.45	(5.9)***	− 1.26	(16.4)***	0.31	(3.6)***	0.36	(1.2)	0.54	(6.5)***	− 1.11	(4.2)***	0.51	(2.8)**
Duration 3	0.04	(0.4)	0.83	(5.5)***	− 1.39	(11.0)***	0.98	(3.4)***	0.47	(1.3)	0.97	(6.9)***	− 1.26	(3.6)***	1.34	(2.4)**
Duration 4	− 0.24	(3.0)**	0.93	(7.0)***	− 1.41	(11.3)***	1.85	(5.1)***	0.03	(0.1)	0.96	(7.9)***	− 1.16	(3.7)***	1.95	(7.0)***
Duration 5	− 0.76	(8.6)***	0.72	(6.1)***	− 1.37	(6.4)***	1.63	(11.9)***	− 0.33	(1.0)	0.74	(7.5)***	− 1.11	(2.2)**	1.64	(7.1)***
Duration 6	− 1.16	(13.7)***	0.36	(3.3)***	− 1.02	(2.6)**	− 3.33	(15.8)***	− 0.56	(1.8)*	0.68	(4.0)***	− 0.21	(0.4)	0.85	(2.4)**
Duration 7	− 1.75	(18.2)***							− 1.21	(4.4)***						
Duration 8	− 2.21	(22.5)***							− 1.61	(4.9)***						
Unobserved heterogeneity																
Constant $(\nu _1, \upsilon _{s,1}, \upsilon _{h,1}, \upsilon _{r,1})$	− 10.63	(20.2)***	− 2.06	(1.9)*	− 4.85	(2.7)**	0.79	(0.5)	− 12.53	(6.6)***	− 2.27	(1.8)*	− 2.59	(0.7)	3.23	(1.3)
$\nu _2$	0.99	(3.6)***							2.04	(5.4)***						
$\upsilon _{s,2}$			− 1.15	(10.3)***							− 1.26	(8.4)***				
$\upsilon _{h,2}$					3.40	(3.2)**							2.70	(2.4)**		
$\upsilon _{r,2}$							0.50	(3.2)**							− 3.29	(8.1)***
$\alpha _1$				0.59	(1.2)							− 0.17	(0.3)			
$\alpha _2$				$-\,\infty$								− 0.52	(1.0)			
$\alpha _{11}$				− 0.30	(0.6)							0.14	(0.2)			
$\alpha _{12}$				$-\,\infty$								− 0.25	(0.2)			
$\alpha _{15}$				− 0.43	(0.3)							$-\,\infty$				
− Log likelihood	215,462.9								18,522.9							
Observations	41,127								3521							

Robust absolute *t* statistics clustered at the HSA level in parentheses

*$$p<0.10$$, **$$p<0.05$$, ***$$p<0.001$$

**Table 10:** Parameter estimates separate processes—post-discharge spending

	Stayers								Movers							
	Mortality		Hospital discharge		In-hospital mortality		Hospital transfer		Mortality		Hospital discharge		In-hospital mortality		Hospital transfer	
	(1)		(2)		(3)		(4)		(5)		(6)		(7)		(8)	
Health-care spending																
In-hospital $(\log T_g)$	− 0.29	(2.0)**	0.21	(2.5)**	− 0.72	(5.2)***	− 0.34	(1.8)*	− 0.92	(1.9)*	0.09	(0.5)	− 1.77	(2.7)**	− 0.35	(1.7)*
Personal characteristics																
Age	0.07	(44.2)***	− 0.01	(16.4)***	0.05	(17.9)***	− 0.02	(11.9)***	0.07	(7.4)***	− 0.01	(5.8)***	0.06	(2.2)**	− 0.02	(2.9)**
Male	0.10	(4.0)***	0.02	(1.7)*	0.02	(0.4)	0.12	(7.1)***	0.05	(0.3)	0.08	(1.8)*	− 0.49	(1.0)	− 0.10	(1.0)
AMI of inferior wall (I21.1)	− 0.21	(4.7)***	0.10	(6.0)***	− 0.17	(2.8)**	0.09	(2.3)**	− 0.12	(0.4)	0.22	(2.7)**	− 0.10	(0.1)	0.19	(1.4)
AMI of other sites (I21.2)	0.00	(0.0)	0.02	(0.6)	− 0.26	(2.9)**	0.00	(0.0)	− 0.45	(1.5)	− 0.15	(1.0)	− 0.72	(0.5)	0.03	(0.1)
AMI of unspecified site (I21.3)	0.20	(3.0)**	− 0.16	(4.5)***	0.29	(2.5)**	− 0.59	(7.5)***	− 0.01	(0.0)	− 0.07	(0.4)	0.80	(0.9)	− 0.78	(1.3)
Non-ST elevation AMI (I21.4)	− 0.10	(2.3)**	− 0.10	(5.6)***	− 1.20	(19.3)***	− 0.22	(3.3)**	− 0.17	(1.1)	− 0.03	(0.3)	− 1.50	(1.5)	− 0.31	(1.6)
AMI, unspecified (I21.9)	0.09	(2.2)**	− 0.23	(6.5)***	0.03	(0.4)	− 0.22	(2.2)**	− 0.29	(1.1)	− 0.24	(1.9)*	− 0.02	(0.0)	− 0.31	(1.2)
Length of stay	0.02	(9.3)***							0.00	(0.1)						
Residence characteristics																
Median income 950€-1010€	− 0.03	(0.7)	0.01	(0.3)	− 0.05	(0.5)	0.14	(1.9)*	0.15	(0.6)	0.04	(0.4)	0.54	(0.8)	0.24	(1.4)
Median income 1010€-1105€	− 0.05	(1.0)	− 0.01	(0.2)	0.04	(0.3)	0.08	(0.7)	0.42	(1.8)*	− 0.03	(0.2)	0.41	(0.6)	− 0.02	(0.1)
Median income >1105€	0.07	(1.4)	− 0.00	(0.0)	0.16	(1.1)	0.38	(2.9)**	0.30	(1.0)	− 0.07	(0.5)	0.69	(1.1)	0.33	(1.5)
University	− 0.01	(3.9)***	− 0.01	(4.1)***	0.01	(1.4)	− 0.02	(2.9)**	− 0.01	(1.3)	0.00	(0.1)	− 0.03	(0.9)	− 0.03	(1.4)
Rural area	− 0.00	(0.1)	− 0.04	(2.8)**	− 0.02	(0.3)	− 0.05	(1.1)	0.37	(1.9)*	− 0.10	(1.2)	− 0.29	(0.7)	− 0.33	(1.4)
Timing																
Weekend	0.07	(2.5)**	0.07	(8.0)***	0.09	(1.9)*	0.17	(8.6)***	− 0.17	(1.0)	− 0.04	(0.7)	0.40	(0.9)	− 0.07	(0.5)
2nd quarter	− 0.00	(0.1)	− 0.02	(1.7)*	− 0.17	(3.6)***	− 0.07	(2.3)**	0.20	(1.0)	0.01	(0.2)	0.29	(0.4)	0.15	(0.9)
3rd quarter	0.03	(1.0)	− 0.02	(1.4)	− 0.08	(1.5)	− 0.05	(2.3)**	− 0.03	(0.1)	− 0.03	(0.4)	0.59	(0.8)	− 0.01	(0.1)
4th quarter	0.02	(0.9)	− 0.01	(0.6)	− 0.07	(1.1)	− 0.01	(0.3)	0.10	(0.5)	0.03	(0.5)	0.33	(0.4)	− 0.14	(0.8)
Comorbidities																
Recent AMI	0.11	(3.9)***	− 0.01	(0.3)	− 0.50	(7.2)***	− 0.90	(17.9)***	0.15	(1.2)	− 0.02	(0.3)	− 0.67	(0.9)	− 0.86	(2.6)**
Cardiovascular disease	0.67	(17.3)***	− 0.15	(10.6)***	0.44	(4.3)***	− 0.26	(4.2)***	0.95	(4.7)***	− 0.11	(0.7)	1.02	(2.3)**	− 0.35	(1.2)
Cerebrovascular disease	0.44	(6.8)***	− 0.14	(4.8)***	0.40	(3.6)***	− 0.25	(3.8)***	0.74	(2.5)**	− 0.19	(2.0)**	− 0.72	(0.6)	− 0.48	(1.2)
Gastrointestinal disease	0.25	(3.7)***	− 0.06	(2.1)**	0.27	(2.3)**	− 0.14	(1.9)*	− 0.44	(0.7)	0.12	(1.0)	0.61	(0.6)	− 1.18	(1.2)
Diabetes	0.42	(15.4)***	− 0.06	(5.0)***	0.17	(2.7)**	0.00	(0.1)	0.27	(1.6)	0.02	(0.3)	0.09	(0.1)	0.00	(0.0)
Respiratory disease	0.28	(6.8)***	− 0.03	(2.2)**	0.28	(4.1)***	− 0.08	(2.3)**	0.09	(0.4)	0.01	(0.1)	1.29	(2.7)**	0.21	(1.3)
Cancer/AIDS	0.53	(12.7)***	− 0.05	(2.7)**	0.21	(3.0)**	− 0.09	(1.6)	1.13	(1.9)*	− 0.12	(0.4)	0.69	(0.7)	− 0.42	(0.9)
Other comorbidities	0.63	(11.4)***	− 0.17	(4.7)***	0.45	(4.2)***	− 0.26	(3.9)***	1.24	(3.5)***	− 0.28	(2.0)**	0.87	(1.3)	− 0.38	(1.2)
Duration dependence																
Constant	− 9.50	(11.9)***	− 2.12	(3.5)***	− 0.78	(5.9)***	1.41	(1.0)	− 7.85	(3.1)**	− 1.45	(1.0)	4.63	(1.0)	1.80	(1.1)
Duration 2	0.26	(2.8)**	0.23	(5.2)***	− 2.42	(2.4)**	− 0.22	(3.8)***	1.61	(1.5)	0.24	(3.8)***	− 1.06	(2.1)**	− 0.36	(2.0)*
Duration 3	0.06	(0.6)	0.41	(6.2)***	− 1.10	(20.1)***	− 0.22	(2.0)**	1.27	(1.2)	0.34	(3.4)***	− 2.00	(2.2)**	− 0.14	(0.6)
Duration 4	− 0.24	(2.9)**	0.34	(7.9)***	− 1.12	(14.3)***	− 0.28	(2.5)**	0.27	(0.2)	0.26	(2.3)**	− 2.01	(1.8)*	− 0.39	(1.2)
Duration 5	− 0.77	(8.6)***	0.09	(2.1)**	− 1.06	(13.8)***	− 0.87	(6.6)***	0.43	(0.5)	− 0.23	(1.4)			− 1.44	(2.3)**
Duration 6	− 1.17	(14.1)***	− 0.27	(4.2)***	− 1.03	(9.1)***	− 2.04	(14.6)***	0.14	(0.1)	0.04	(0.2)			− 1.27	(1.6)
Duration 7	− 1.79	(21.0)***							− 0.42	(0.4)						
Duration 8	− 2.30	(30.3)***							− 0.65	(0.7)						
− Log likelihood	230,457.8								5579.5							
Observations	43,510								1138							

Robust absolute *t* statistics clustered at the HSA level in parentheses. Duration dependence parameters for durations 5 and 6 not estimated due to insufficient number of events in movers’ in-hospital mortality equation

*$$p<0.10$$, **$$p<0.05$$, ***$$p<0.001$$

**Table 11:** Parameter estimates correlated model—post-discharge spending

	Stayers								Movers							
	Mortality		Hospital discharge		In-hospital mortality		Hospital transfer		Mortality		Hospital discharge		In-hospital mortality		Hospital transfer	
	(1)		(2)		(3)		(4)		(5)		(6)		(7)		(8)	
Health-care spending																
Post-discharge $(\log T_g^P)$	− 0.28	(3.1)**	0.28	(15.4)***	− 0.72	(9.3)***	− 0.12	(3.0)**	− 0.97	(1.9)*	0.20	(1.0)	− 1.79	(2.6)**	− 0.11	(0.3)
Personal Characteristics																
Age	0.07	(52.0)***	− 0.01	(27.6)***	0.05	(22.0)***	− 0.02	(17.8)***	0.07	(6.9)***	− 0.01	(4.3)***	0.06	(2.1)**	− 0.01	(1.9)*
Male	0.10	(4.4)***	0.01	(0.8)	0.03	(0.6)	0.09	(3.4)***	0.02	(0.1)	0.12	(2.1)**	− 0.48	(1.0)	0.02	(0.1)
AMI of inferior wall (I21.1)	− 0.22	(6.2)***	0.11	(6.7)***	− 0.16	(2.4)**	0.12	(3.6)***	− 0.14	(0.5)	0.24	(2.5)**	− 0.10	(0.1)	0.23	(1.1)
AMI of other sites (I21.2)	− 0.02	(0.3)	0.01	(0.4)	− 0.28	(2.3)**	− 0.01	(0.2)	− 0.48	(1.3)	− 0.13	(0.5)	− 0.78	(0.6)	− 0.02	(0.0)
AMI of unspecified site (I21.3)	0.22	(3.6)***	− 0.22	(6.2)***	0.31	(2.9)**	− 0.79	(8.5)***	− 0.03	(0.1)	− 0.27	(1.1)	0.91	(1.0)	− 1.39	(3.0)**
Non-ST elevation AMI (I21.4)	− 0.14	(4.3)***	− 0.15	(9.5)***	− 1.21	(16.5)***	− 0.35	(10.4)***	− 0.17	(1.0)	− 0.12	(1.1)	− 1.54	(1.6)	− 0.63	(3.0)**
AMI, unspecified (I21.9)	0.09	(2.4)**	− 0.30	(14.9)***	0.05	(0.8)	− 0.43	(9.7)***	− 0.30	(1.1)	− 0.37	(2.8)**	− 0.07	(0.1)	− 0.78	(2.7)**
Length of stay	0.02	(8.2)***							− 0.01	(0.3)						
Residence characteristics																
Median income 950€-1010€	− 0.02	(0.7)	− 0.01	(0.6)	− 0.04	(0.5)	0.09	(2.5)**	0.18	(0.7)	0.04	(0.4)	0.57	(0.8)	0.31	(1.4)
Median income 1010€-1105€	− 0.05	(1.3)	− 0.02	(1.5)	0.04	(0.5)	0.03	(0.9)	0.46	(1.8)*	− 0.08	(0.6)	0.43	(0.7)	− 0.17	(0.5)
Median income >1105€	0.08	(1.8)*	− 0.02	(1.0)	0.17	(1.9)*	0.32	(7.1)***	0.34	(1.1)	− 0.07	(0.4)	0.80	(1.3)	0.41	(1.1)
University	− 0.01	(4.6)***	− 0.01	(8.5)***	0.01	(1.8)*	− 0.03	(10.1)***	− 0.02	(1.5)	− 0.00	(0.1)	− 0.03	(1.0)	− 0.03	(1.5)
Rural area	− 0.01	(0.3)	− 0.06	(3.7)***	− 0.02	(0.3)	− 0.11	(3.3)***	0.39	(1.9)*	− 0.09	(1.1)	− 0.33	(0.7)	− 0.30	(1.2)
Timing																
Weekend	0.09	(3.3)***	0.05	(4.0)***	0.13	(2.4)**	0.13	(4.7)***	− 0.16	(1.0)	− 0.08	(1.1)	0.34	(0.8)	− 0.18	(0.9)
2nd quarter	− 0.01	(0.3)	− 0.02	(1.5)	− 0.18	(2.7)**	− 0.08	(2.3)**	0.21	(0.9)	0.01	(0.1)	0.31	(0.4)	0.11	(0.5)
3rd quarter	0.03	(1.1)	− 0.03	(1.8)*	− 0.07	(1.1)	− 0.08	(2.3)**	− 0.01	(0.0)	− 0.03	(0.3)	0.55	(0.7)	− 0.02	(0.1)
4th quarter	0.02	(0.8)	− 0.02	(1.1)	− 0.07	(1.1)	− 0.03	(0.8)	0.13	(0.6)	− 0.01	(0.2)	0.29	(0.3)	− 0.28	(2.0)**
Comorbidities																
Recent AMI	0.10	(3.6)***	− 0.00	(0.1)	− 0.51	(7.1)***	− 0.88	(21.0)***	0.15	(1.1)	0.01	(0.2)	− 0.70	(0.9)	− 0.74	(1.8)*
Cardiovascular disease	0.72	(20.2)***	− 0.15	(6.0)***	0.47	(6.3)***	− 0.25	(4.1)***	0.98	(4.6)***	− 0.05	(0.3)	1.16	(2.0)**	− 0.20	(0.5)
Cerebrovascular disease	0.47	(8.8)***	− 0.16	(4.2)***	0.41	(3.9)***	− 0.30	(3.2)**	0.78	(2.5)**	− 0.20	(1.8)*	− 0.83	(0.7)	− 0.47	(0.9)
Gastrointestinal disease	0.27	(4.2)***	− 0.07	(1.8)*	0.28	(2.2)**	− 0.16	(1.7)*	− 0.48	(0.7)	0.04	(0.2)	0.54	(0.5)	− 1.37	(1.1)
Diabetes	0.44	(18.2)***	− 0.06	(4.7)***	0.18	(3.5)***	− 0.01	(0.4)	0.28	(1.6)	0.01	(0.1)	0.06	(0.1)	0.07	(0.4)
Respiratory disease	0.30	(8.4)***	− 0.04	(1.7)*	0.29	(3.9)***	− 0.08	(1.6)*	0.12	(0.5)	− 0.06	(0.5)	1.22	(2.5)**	− 0.05	(0.2)
Cancer/AIDS	0.57	(13.3)***	− 0.04	(1.4)	0.19	(1.9)*	− 0.04	(0.6)	1.19	(1.8)*	− 0.15	(0.6)	0.62	(0.6)	− 0.46	(1.0)
Other comorbidities	0.68	(15.4)***	− 0.19	(5.7)***	0.46	(5.1)***	− 0.32	(4.0)***	1.32	(3.9)***	− 0.39	(1.8)*	0.84	(1.2)	− 0.82	(1.6)
Duration dependence																
Duration 2	0.26	(2.7)**	0.46	(32.7)***	− 1.27	(18.6)***	0.32	(11.6)***	1.62	(1.5)	0.38	(6.0)***	− 1.02	(2.1)**	0.01	(0.1)
Duration 3	0.07	(0.7)	0.84	(44.6)***	− 1.41	(16.3)***	0.99	(21.0)***	1.28	(1.2)	0.63	(6.2)***	− 1.92	(2.0)**	0.70	(2.8)**
Duration 4	− 0.22	(2.7)**	0.94	(44.5)***	− 1.43	(15.5)***	1.86	(28.6)***	0.28	(0.2)	0.72	(5.8)***	− 1.88	(1.7)*	1.23	(3.3)***
Duration 5	− 0.74	(8.8)***	0.73	(24.8)***	− 1.40	(10.4)***	1.64	(18.3)***	0.46	(0.5)	0.36	(1.8)*			1.64	(2.2)**
Duration 6	− 1.13	(13.8)***	0.38	(9.8)***	− 1.06	(6.5)***	− 3.32	(63.2)***	0.19	(0.2)	0.66	(2.9)**			2.35	(1.8)*
Duration 7	− 1.73	(21.9)***							− 0.34	(0.3)						
Duration 8	− 2.19	(27.6)***							− 0.49	(0.5)						
Unobserved heterogeneity																
Constant $(\nu _1, \upsilon _{s,1}, \upsilon _{h,1}, \upsilon _{r,1})$	− 10.09	(19.8)***	− 2.02	(14.1)***	− 5.29	(4.4)***	1.03	(3.3)***	− 7.98	(3.0)**	− 1.96	(1.2)	4.84	(0.9)	0.56	(0.2)
$\nu _2$	1.10	(7.0)***							1.40	(3.3)***						
$\upsilon _{s,2}$			− 1.16	(65.3)***							− 0.96	(10.1)***				
$\upsilon _{h,2}$					3.65	(3.5)***							$-\,\infty$			
$\upsilon _{r,2}$							0.49	(3.1)**							− 4.36	(3.4)***
$\alpha _1$				0.72	(3.6)***							1.77	(2.4)**			
$\alpha _{11}$				− 0.54	(1.2)							1.18	(1.1)			
$\alpha _{15}$				0.22	(0.5)							$-\,\infty$				
− Log likelihood	228,517.8								5536.6							
Observations	43,510								1138							

Robust absolute *t* statistics clustered at the HSA level in parentheses. Duration dependence parameters for durations 5 and 6 not estimated due to insufficient number of events in movers’ in-hospital mortality equation

*$$p<0.10$$, **$$p<0.05$$, ***$$p<0.001$$

**Table 12:** Descriptive statistics by quartile of spending intensity for locals/visitors

Patient group	Locals				Visitors			
	Q1	Q2	Q3	Q4	Q1	Q2	Q3	Q4
Health-care spending								
In-hospital ($\log T_g$)	7.144	7.467*	7.809*	8.017*	7.176	7.444*	7.744*	8.019*
Outcome								
In-hospital mortality	0.072	0.042	0.025*	0.043*	0.091	0.046*	0.036*	0.042*
Post-discharge mortality	0.308	0.247	0.233*	0.228*	0.300	0.269	0.267	0.237*
vObserved duration^a^	700.6	771.8	847.0*	776.3*	616.2	733.7*	752.1*	685.0*
Length of stay^a^	5.8	5.8	5.1	5.1	6.2	6.2	5.6	5.1*
Personal characteristics								
Age	69.0	67.5	66.5*	66.2*	67.6	66.2	65.8	63.7*
Male	0.565	0.623*	0.638*	0.661*	0.608	0.618	0.648	0.670
Log of 1-year costs prior to AMI	6.868	6.715	6.782	6.585*	6.704	6.581	6.811	6.678
AMI of anterior wall (I21.0)	0.192	0.221	0.208	0.278*	0.201	0.208	0.223	0.266
AMI of inferior wall (I21.1)	0.183	0.233	0.232	0.290	0.176	0.185	0.164	0.299
AMI of other sites (I21.2)	0.039	0.050	0.051	0.039	0.028	0.029	0.020	0.036
AMI of unspecified site (I21.3)	0.017	0.020	0.066*	0.017	0.022	0.027	0.066*	0.011
Non-ST elevation AMI (I21.4)	0.388	0.307	0.346	0.273*	0.337	0.356	0.434	0.303
AMI, unspecified (I21.9)	0.180	0.168	0.097	0.102	0.235	0.195	0.093*	0.085*
Residence characteristics								
Median income	967.5	1012.7	1020.0*	1123.7	1001.5	1018.0	975.5	963.6
University educated	0.129	0.141	0.151*	0.199*	0.145	0.139	0.129	0.112*
Rural area	0.310	0.316	0.247*	0.193*	0.252	0.350	0.252	0.454*
Comorbidities								
Charlson index	0.998	0.879	0.959	0.811*	0.980	0.873	1.091	0.978
Recent AMI	0.160	0.144	0.208*	0.169	0.171	0.134	0.244	0.216
Cardiovascular disease	0.068	0.053	0.068	0.050*	0.078	0.069	0.109*	0.058
Cerebrovascular disease	0.032	0.022*	0.025	0.018*	0.030	0.034	0.036	0.029
Gastrointestinal disease	0.023	0.020	0.017	0.020	0.018	0.015	0.013	0.019
Diabetes	0.262	0.239	0.242	0.221*	0.263	0.226	0.244	0.238
Respiratory disease	0.082	0.076	0.079	0.065*	0.083	0.081	0.086	0.076
Cancer/AIDS	0.049	0.043	0.042	0.040*	0.036	0.047	0.041	0.042
Other comorbidities	0.044	0.029*	0.037	0.027*	0.042	0.027	0.039	0.041
Timing								
Weekend	0.236	0.229	0.213*	0.235	0.257	0.260	0.215*	0.222*
1st quarter	0.241	0.234	0.232	0.228*	0.245	0.212	0.233	0.211
2nd quarter	0.234	0.248*	0.240	0.239	0.237	0.253	0.250	0.240
3rd quarter	0.252	0.250	0.261	0.254	0.249	0.266	0.258	0.239
4th quarter	0.273	0.268	0.267	0.279	0.269	0.269	0.259	0.309*
Observations	10,840	10,227	9879	10,181	919	655	861	1086

*Significantly different from bottom quartile at the 5-percent level. Standard errors clustered at the HSA level

^a^Reported in days

**Table 13:** Descriptive statistics by quartile of spending intensity for movers/stayers

Patient group	Stayers				Movers			
	Q1	Q2	Q3	Q4	Q1	Q2	Q3	Q4
Health-care spending								
Post-discharge ($\log T^P_g$)	5.434	5.624*	5.716*	5.790*	5.446	5.623*	5.719*	5.789*
Outcome								
In-hospital mortality	0.043	0.054	0.054	0.034	0.023	0.022	0.017	0.027
Post-discharge mortality	0.254	0.274	0.258	0.236	0.203	0.196	0.182	0.203
Observed duration^a^	767.6	750.9	759.6	791.6	783.5	797.2	838.9	766.3
Length of stay^a^	5.3	5.6	5.6	5.4	5.3	5.5	5.9	5.8
Personal characteristics								
Age	66.8	67.6	67.8*	66.8	64.1	62.9	65.6	64.0
Male	0.605	0.615	0.637	0.636	0.641	0.717	0.600	0.649
Log of 1-year costs prior to AMI	6.747	6.786	6.814	6.831	6.931	6.752	6.839	6.706
AMI of anterior wall (I21.0)	0.206	0.224	0.238	0.236	0.188	0.246	0.249*	0.257
AMI of inferior wall (I21.1)	0.219	0.229	0.241	0.245	0.219	0.227	0.225	0.284
AMI of other sites (I21.2)	0.034	0.052	0.040	0.053	0.055	0.050	0.039	0.027
AMI of unspecified site (I21.3)	0.033	0.027	0.025	0.035	0.031	0.031	0.034	0.020
Non-ST elevation AMI (I21.4)	0.392	0.334	0.303*	0.274*	0.418	0.299*	0.269*	0.277*
AMI, unspecified (I21.9)	0.116	0.134	0.154	0.157	0.090	0.146	0.184*	0.135
Residence characteristics								
Median income	956.3	1003.4	1121.1*	1022.6*	961.6	993.7	1153.6*	1040.1*
University	0.142	0.136	0.184	0.144	0.143	0.132	0.203*	0.142
Rural area	0.268	0.308	0.198*	0.342*	0.281	0.361	0.194*	0.365
Comorbidities								
Charlson index	0.936	0.960	0.888	0.894	0.816	0.692	0.799	0.872
Recent AMI	0.159	0.185	0.166	0.180	0.176	0.168	0.199	0.135
Cardiovascular disease	0.073	0.063	0.056*	0.049*	0.078	0.040	0.044	0.054
Cerebrovascular disease	0.028	0.027	0.022*	0.021*	0.043	0.022	0.036	0.020
Gastrointestinal disease	0.018	0.019	0.018	0.031*	0.008	0.019	0.007	0.020
Diabetes	0.247	0.240	0.242	0.237	0.195	0.193	0.223	0.270
Respiratory disease	0.092	0.075*	0.063*	0.076	0.082	0.047	0.044*	0.034*
Cancer/AIDS	0.040	0.050*	0.041	0.046	0.016	0.006	0.027	0.041
Other comorbidities	0.035	0.032	0.034	0.040	0.031	0.034	0.031	0.034
Timing								
Weekend	0.232	0.228	0.227	0.227	0.262	0.243	0.237	0.236
1st quarter	0.235	0.236	0.233	0.227	0.242	0.224	0.218	0.236
2nd quarter	0.235	0.241	0.241	0.244	0.246	0.249	0.269	0.230
3rd quarter	0.253	0.256	0.252	0.257	0.227	0.268	0.220	0.284
4th quarter	0.277	0.267	0.273	0.272	0.285	0.259	0.293	0.250
Observations	12,390	11,819	12,014	7287	256	321	413	148

* Significantly different from bottom quartile at the 5-percent level. Standard errors clustered at the HSA level

^a^Reported in days

## Appendix 3

### Magnitude of effects

For an exponential MPH model, the survivor function *S* is given by $S(s \mid x,t,u)= \exp (-\int _0^t \theta (s \mid x,t,u) \, ds$, with $\theta = \lambda (t) \exp (x'\beta +u)$, where $\lambda (t)$ denotes the duration dependence. The predicted survival probabilities are then obtained by calculating the linear predictor $x'\beta$ using the estimated coefficients (including the duration dependence) set at given values of explanatory variables and averaged over the unobserved heterogeneity distribution. For spending effects, we assume a median-aged 67-year old male (sample median), hospitalized for 5 days (sample median) with ST elevation myocardial infarction of anterior wall during the first quarter of year, with two most common comorbidities—diabetes and and cardiovascular disease. We also assume that this individual neither had a PCI intervention nor a surgery and was not hospitalized during weekend. The residence of this patient was in metropolitan area and in the highest income bracket. The spending predictor is then varied based on percentiles of spending distribution. The same patient is then assumed for the simulation of the age effects, except that the age is varied based on percentiles of the age distribution, while spending is fixed at a median-spending HSA.

## Appendix 4

### Districts in Slovakia

See Table 14.

**Table 14:** Districts in Slovakia

District	Code	Region	Population	Area (km^2^)	Population density	Number of municipalities
Bánovce nad Bebravou	BN	Trenčiansky	35,658	461.9	77	43
Banská Bystrica	BB	Banskobystrický	108,120	809.4	134	42
Banská štiavnica	BS	Banskobystrický	15,551	292.3	53	15
Bardejov	BJ	Prešovský	75,786	936.2	81	86
Bratislava I	BA I	Bratislavský	46,432	9.6	4842	
Bratislava II	BA II	Bratislavský	125,001	92.5	1352	
Bratislava III	BA III	Bratislavský	76,694	74.7	1027	
Bratislava IV	BA IV	Bratislavský	105,154	96.7	1087	
Bratislava V	BA V	Bratislavský	122,296	94.2	1298	
Brezno	BR	Banskobystrický	58,965	1265.2	47	30
Bytča	BY	Žilinský	31,163	281.6	111	12
Čadca	CA	Žilinský	87,969	760.6	116	23
Detva	DT	Banskobystrický	30,854	449.2	69	15
Dolný Kubín	DK	Žilinský	38,937	491.9	79	24
Dunajská Streda	DS	Trnavský	125,238	1074.6	117	67
Galanta	GA	Trnavský	95,027	641.7	148	36
Gelnica	GL	Košický	31,668	584.4	54	20
Hlohovec	HC	Trnavský	43,769	267.2	164	24
Humenné	HE	Prešovský	59,535	754.2	79	62
Ilava	IL	Trenčiansky	57,511	358.5	160	21
KeŽmarok	KK	Prešovský	74,232	839.3	88	41
Komárno	KN	Nitriansky	100,212	1100.1	91	41
Košice I	KE I	Košický	63,904	85.4	748	
Košice II	KE II	Košický	79,034	73.9	1070	
Košice III	KE III	Košický	27,924	16.9	1657	
Košice IV	KE IV	Košický	56,596	60.9	929	
Košice-okolie	KS	Košický	129,237	1541.3	84	114
Krupina	KA	Banskobystrický	21,366	584.9	37	36
Kysucké Nové Mesto	KM	Žilinský	32,654	173.7	188	14
Levice	LV	Nitriansky	109,588	1551.1	71	89
Levoča	LE	Prešovský	33,127	357.3	93	33
Liptovský Mikuláš	LM	Žilinský	71,685	1341.1	53	56
Lučenec	LC	Banskobystrický	69,788	825.6	85	57
Malacky	MA	Bratislavský	78,809	949.5	83	26
Martin	MT	Žilinský	93,816	735.7	128	43
Medzilaborce	ML	Prešovský	10,870	427.3	25	23
Michalovce	MI	Košický	108,520	1019.3	106	78
Myjava	MY	Trenčiansky	25,363	327.4	77	17
Námestovo	NO	Žilinský	63,563	690.5	92	24
Nitra	NR	Nitriansky	164,580	870.7	189	62
Nové Mesto nad Váhom	NM	Trenčiansky	61,512	580.0	106	34
Nové Zámky	NZ	Nitriansky	137,001	1347.1	102	62
Partizánske	PE	Trenčiansky	44,179	301.0	147	23
Pezinok	PK	Bratislavský	69,623	375.5	185	17
Piešt’any	PN	Trnavský	62,662	381.1	164	27
Poltár	PT	Banskobystrický	20,427	476.2	43	22
Poprad	PP	Prešovský	102,469	1105.4	93	29
PovaŽská Bystrica	PB	Trenčiansky	61,211	463.2	132	28
Prešov	PO	Prešovský	173,187	933.7	185	91
Prievidza	PD	Trenčiansky	130,616	959.8	136	52
Púchov	PU	Trenčiansky	44,133	375.3	118	21
Revúca	RA	Banskobystrický	38,242	730.3	52	42
Rimavská Sobota	RS	Banskobystrický	80,359	1471.1	55	107
RoŽňava	RV	Košický	58,995	1173.3	50	62
RuŽomberok	RK	Žilinský	57,036	646.8	88	25
Sabinov	SB	Prešovský	60,607	483.5	125	43
Senec	SC	Bratislavský	99,705	359.9	277	29
Senica	SE	Trnavský	59,347	683.5	87	31
Skalica	SI	Trnavský	47,313	357.1	132	21
Snina	SV	Prešovský	34,655	804.7	43	34
Sobrance	SO	Košický	22,377	538.2	42	47
Spišská Nová Ves	SN	Košický	98,656	587.4	168	36
Stará Ĺubovňa	SL	Prešovský	52,867	624.0	85	44
Stropkov	SP	Prešovský	19,744	389.0	51	43
Svidník	SK	Prešovský	31,397	549.8	57	68
šaía	SA	Nitriansky	50,972	355.9	143	13
Topoíčany	TO	Nitriansky	70,313	597.6	118	54
Trebišov	TV	Košický	103,377	1073.5	96	82
Trenčín	TN	Trenčiansky	113,516	674.8	168	37
Trnava	TT	Trnavský	131,940	741.3	178	45
Turčianske Teplice	TR	Žilinský	15,848	392.8	40	26
Tvrdošín	TS	Žilinský	35,802	478.9	75	15
Veíký Krtíš	VK	Banskobystrický	41,605	848.2	49	71
Vranov nad Topíou	VT	Prešovský	79,181	769.5	103	68
Zlaté Moravce	ZM	Nitriansky	40,881	521.2	78	33
Zvolen	ZV	Banskobystrický	66,294	759.0	87	26
Žarnovica	ZC	Banskobystrický	24,991	425.3	59	18
Žiar nad Hronom	ZH	Banskobystrický	44,424	517.7	86	35
Žilina	ZA	Žilinský	161,052	815.1	198	53

Based on data from the Statistical Office of the Slovak Republic

## Footnotes

1 Heart attacks occur when there is a blockage in the supply of blood to the heart causing the heart muscle to receive insufficient oxygen. The blockage is typically caused by a blood clot. Reperfusion—restoration of the blood flow to the heart is urgent. Blood clots can be broken down by pharmacological means or angioplasty where a catheter balloon is inflated inside the blocked coronary artery.

2 Health care is expensive. Despite a slowdown following the recessions in 2008 and 2013, annual per capita health spending growth across the Organization for Economic Co-operation and Development (OECD) countries has been around 2.4%. In absolute terms, this translates to an overall average for all member countries of around $4000. Over the same period, the 30-day mortality rates for AMI decreased from 12.5 to 9.1% in OECD countries [1].

3 Heart attacks are also frequently studied because the health outcome (mortality) is easily measured and often occurs when the patient is still in hospital.

4 Health-care utilization is defined as a sum of all costs, including inpatient and outpatient care, pharmaceutical prescriptions, emergency care etc. accrued by patients during a calendar year, based on their resident HSA. Appendix Table 14 provides an overview of all two-letter districts and their full names.

5 For more information about the Slovak health-care system, see for example Bucek Psenkova et al. [11].

6 Costs are determined by DRGs, where the hospital base rate is multiplied by relative weights. The base rate is set by the Health Care Surveillance Authority, a bureau responsible for overlooking health care provided by public health care insurance.

7 Despite the fact the databases allow us to track patients up to the end of year 2021, we decided to limit the observation window up to the end of year 2019 due to disruptions in delivery of health care during the COVID-19 pandemic. In the sensitivity analysis section we also consider a longer observation window in the analysis of post-discharge survival.

8 PCI are minimally invasive procedures used to open clogged coronary arteries—those that deliver blood to the heart. Standard treatment guidelines for heart attacks recommend a transport of patients into a PCI center within 90 min since confirmed diagnosis of ST-elevation AMI. This is also the case in Slovakia, by recommendation of the official guidelines published by the Ministry of Health.

9 A study by Barlík et al. [14] analyzing the number of active SIM cards in the capital of Slovakia shows that during weekdays, the number of active SIM cards during business hours is almost 20% higher than during weekends.

10 The event study equation is defined as follows: $y_{it} = \alpha _i + \sum _{k=-6}^{6} \beta _k 1(t-t^*_i=k) + \psi _t + \epsilon _{it}$, where $y_{it}$ captures log of yearly health care utilization, $\alpha _i$ are individual fixed effects, $t^*_i$ denotes year of move of individual *i*, $\phi _t$ captures calendar-year effects. The main event study coefficients of interest are denoted as $\beta _k$ for $k \in [-6,6]$ and are identified relative to each other. We normalize the pre-move year $\beta _{-1} = 0$.

11 Visitors may be relocated from a hospital in the region they visited to a hospital in the region of residence. For visitors, we use the health-care expenditures of the first hospital they entered after their heart attack.

12 This phenomenon is not unique to Slovakia. For example, Karlson et al. [16] find a similar peak in the post-discharge mortality rate for Swedish AMI patients.

13 In general, non-ST-elevated AMI is caused by a partial blockage of coronary arteries. ST-elevated AMI is caused by a full blockage and therefore carries higher risk of complications and death.

14 We include the first-hospital treatment intensity since the crucial treatment decisions determining survival of patients such as whether or not to administer reperfusion therapy or PCI are time-dependent and are unlikely to be performed after more than 24–48 h since the diagnosis (O’Gara et al. [18]).

15 The quarter of year is included, since there is a well-documented seasonality of cardiovascular diseases, with greater incidence and mortality observed during winter months. Furthermore, concurrent influenza and increase in air pollution during winter are likely contributors to worse health outcomes (Stewart et al. [19]).

16 The full set of estimates for columns 1–4 are presented in Appendix Tables 8 and 9. These appendix tables also contain the parameter estimates for the hospital discharge and transfer rates.

17 Note that a power calculation to find a minimum effect of 0.5 for a similar Cox proportional hazards model, assuming power of 0.8, significance of 0.10 and event probability equal to 0.20 (sample post-discharge mortality) requires minimum sample size of $N = 495$ and a minimum number of events 99.

18 Appendix 1 presents a straightforward and simple analysis using linear probability models for in-hospital and post-discharge mortality of patients who suffered from a heart attack. The results suggest that mortality of both locals and visitors are affected by personal characteristics and residence characteristics. In-hospital health-care expenditures have a significant negative effect on the probability to die in-hospital. Post-discharge health-care expenditures reduce mortality after the discharge from hospital.

19 Predicted survival probabilities for females are similar to those of males.

20 While our dataset includes detailed information about post-discharge procedures, it does not contain detailed information about in-hospital procedures, except whether a bypass surgery or a PCI intervention was performed. See for example Doyle [7] for a disaggregation of in-hospital procedures of AMI patients and their relationship with health-care spending.

21 Cardiac rehabilitation is a customized outpatient program of exercise and education. The program is designed to help improve health and stimulate recovery from a heart attack. In Slovakia, this often includes spa cardiac rehabilitation.
